# SIRT7‐Mediated H2BK120 Succinylation Drives Aberrant Mitophagy in Sepsis‐Associated Cognitive Dysfunction

**DOI:** 10.1002/advs.77642

**Published:** 2026-09-08

**Authors:** Na Meng, Jiateng Zhou, Xiaoyu Guo, Ting Hong, Xuelian Li, Xingyu Wei, Chanhua Zhang, Xixue Zhang, Songbin Liu, Yulin Zhang, Liangfang Yao, Lina Huang, Weidong Gu

**Affiliations:** ^1^ Department of Anesthesiology, Huadong Hospital Fudan University Shanghai China; ^2^ Department of Dermatology, Shanghai Tongji Hospital Tongji University School of Medicine Shanghai China; ^3^ Department of Anesthesiology, Shanghai General Hospital Shanghai Jiao Tong University Shanghai China

**Keywords:** citric acid cycle, epigenetics, histone, mitochondrion, mitophagy, SIRT7, succinylation

## Abstract

Sepsis‐associated encephalopathy (SAE) is a severe neurological complication of sepsis, yet how metabolic disturbances engage epigenetic regulation in SAE remains unclear. We found that septic mice exhibited hippocampal succinate and succinyl‐CoA accumulation, accompanied by enhanced neuronal histone H2BK120 succinylation (H2BK120su). Pharmacological reduction of succinylation alleviated neuronal injury and improved cognitive function. Mechanistically, integrated CUT&Tag and transcriptomic analyses identified *Pdcd1* as a downstream gene associated with H2BK120su enrichment. H2BK120su enrichment at the *Pdcd1* promoter activated the PD‐1/PD‐L1 axis, promoted mitochondrial translocation of PD‐L1 and its interaction with PINK1, and triggered PINK1/Parkin‐dependent mitophagy, leading to mitochondrial dysfunction and neuronal apoptosis. Neutralization of PD‐1/PD‐L1 or knockdown of *Pdcd1* attenuated mitophagy and neuronal injury. We further identified SIRT7 downregulation as a major cause of H2BK120su accumulation in the septic hippocampus. Neuron‐specific *Sirt7* deletion exacerbated H2BK120su enrichment, PD‐1/PD‐L1 activation, excessive mitophagy, and cognitive impairment, whereas SIRT7 overexpression reversed these pathological changes. Together, our findings define a SIRT7‐H2BK120su‐PD‐1/PD‐L1‐PINK1 axis linking metabolic reprogramming to aberrant mitophagy in SAE and suggest SIRT7‐dependent succinylation as a potential therapeutic target.

## Graphical abstract

Sepsis triggers hippocampal SIRT7 loss, promoting histone H2B succinylation and activation of the PD‐1/PD‐L1–PINK1 axis. This metabolic‐epigenetic cascade drives aberrant mitophagy and neuronal injury, ultimately causing cognitive deficits. Targeting SIRT7‐dependent succinylation offers a potential strategy to protect brain function during sepsis.

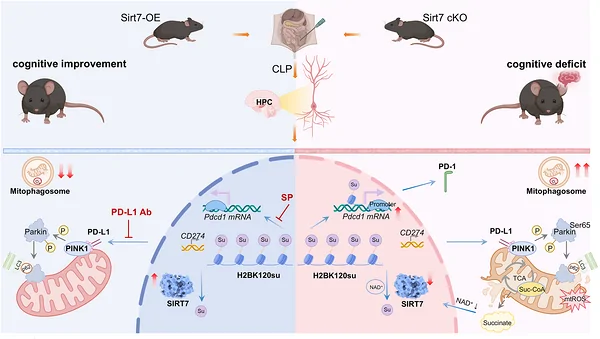

## 1 Introduction

Sepsis is a life‐threatening systemic inflammatory response to infection that frequently leads to multi‐organ dysfunction. Among its severe complications, sepsis‐associated encephalopathy (SAE) represents a diffuse cerebral dysfunction independently associated with high mortality and long‐term cognitive impairment in survivors [1, 2, 3, 4]. Although the exact pathogenesis of SAE remains elusive, emerging evidence underscores the pivotal role of metabolic reprogramming and its dynamic interplay with post‐translational modifications (PTMs) in driving sepsis‐induced brain injury. These PTMs, which are highly sensitive to cellular metabolic states, have been shown to critically regulate key biological processes such as mitochondrial function and epigenetic modulation in various disease contexts [5, 6]. Elucidating the mechanisms by which metabolism‐driven post‐translational modifications lead to neuronal injury during SAE progression holds significant importance for identifying potential therapeutic targets.

Lysine succinylation, a reversible post‐translational modification, has attracted significant attention for its role in coupling cellular metabolism to epigenetic regulation. This modification, driven by the metabolic intermediate succinyl‐CoA, can induce substantial structural and functional alterations in substrate proteins and is particularly enriched on mitochondrial proteins and core histones [7, 8, 9]. The dynamic balance of succinylation is maintained by “writer” enzymes (e.g., KAT2A) and “eraser” deacylases, among which the sirtuin family members SIRT5 and SIRT7 are of particular importance [10, 11]. As a nuclear‐localized deacylase, SIRT7 plays a critical role in maintaining genomic stability, regulating metabolic processes, and influencing disease progression. Disruption of lysine succinylation homeostasis has been implicated in various pathological processes, including sepsis‐induced organ dysfunction. However, its specific role and regulatory mechanisms in SAE, particularly whether SIRT7 serves as a central regulator in this process, remain to be elucidated [12, 13].

In this study, we demonstrated that SIRT7 expression, rather than SIRT5, was markedly downregulated in hippocampal neurons during SAE. Mechanistically, diminished SIRT7 activity led to the accumulation of histone H2B lysine 120 succinylation (H2BK120su) at the promoter region of Pdcd1, thereby driving aberrant activation of the PD‐1/PD‐L1 immune checkpoint axis. This activation prompted PD‐L1 translocation to mitochondria, where it interacted with PINK1 and induced excessive PINK1/Parkin‐dependent mitophagy, ultimately culminating in neuronal death and cognitive deficits. Notably, restoration of SIRT7 function or pharmacological inhibition of succinylation effectively attenuated these pathological alterations. Our findings identified a previously unrecognized SIRT7/H2BK120su/PD‐1/PD‐L1 regulatory axis in sepsis‐induced neuronal injury, revealing a potential therapeutic target for SAE.

## 2 Results

### 2.1 Metabolomic Profiling of the Hippocampus in SAE Reveals Disruption of Amino Acid and Nucleotide Metabolism

To obtain an integrated view of metabolic reprogramming during hippocampal injury in SAE mice, we first performed an untargeted metabolomic analysis using liquid chromatography‐mass spectrometry (LC‐MS) on hippocampal tissues from sham‐operated and cecal ligation and puncture (CLP) induced septic mice. To ensure data reliability, quality control (QC) samples were analyzed throughout the acquisition sequence. The total ion chromatograms (TICs) of QC samples showed substantial overlap in peak intensities and retention times, with no apparent signal drift or alterations in peak shape, indicating good instrument stability throughout the analytical run (Figure S1a). Principal component analysis (PCA) further demonstrated tight clustering of QC samples, supporting robust reproducibility of the LC‐MS measurements (Figure S1b).

A total of 1,709 metabolites were identified (Figure S1c), spanning multiple chemical classes, including carboxylic acids and their derivatives, fatty acyls, glycerophospholipids, and related metabolites (Figure S1d). PCA performed on the annotated metabolite features revealed a clear separation between the septic and sham groups, indicating distinct metabolic profiles (Figure S1e). Using univariate analysis (P < 0.05 and |log2FC| > 1.5), we identified 599 significantly altered metabolites in septic mice, including 177 upregulated and 422 downregulated species (Figure S1f). Subsequently, pathway enrichment analysis of these differential metabolites showed predominant enrichment in amino acid and nucleotide metabolism, cofactor‐related pathways, and redox‐associated processes, such as biosynthesis of amino acids, riboflavin metabolism, glutathione metabolism, and 2‐oxocarboxylic acid metabolism (Figure S1g). Collectively, these results indicate that there is a profound disruption of hippocampal amino acid and nucleotide pools in septic mice, which may secondarily impair mitochondrial bioenergetics and redox balance.

### 2.2 Hippocampal Metabolic Perturbations are Associated with Neuron‐Specific H2BK120 Succinylation in SAE

**FIGURE 1:**
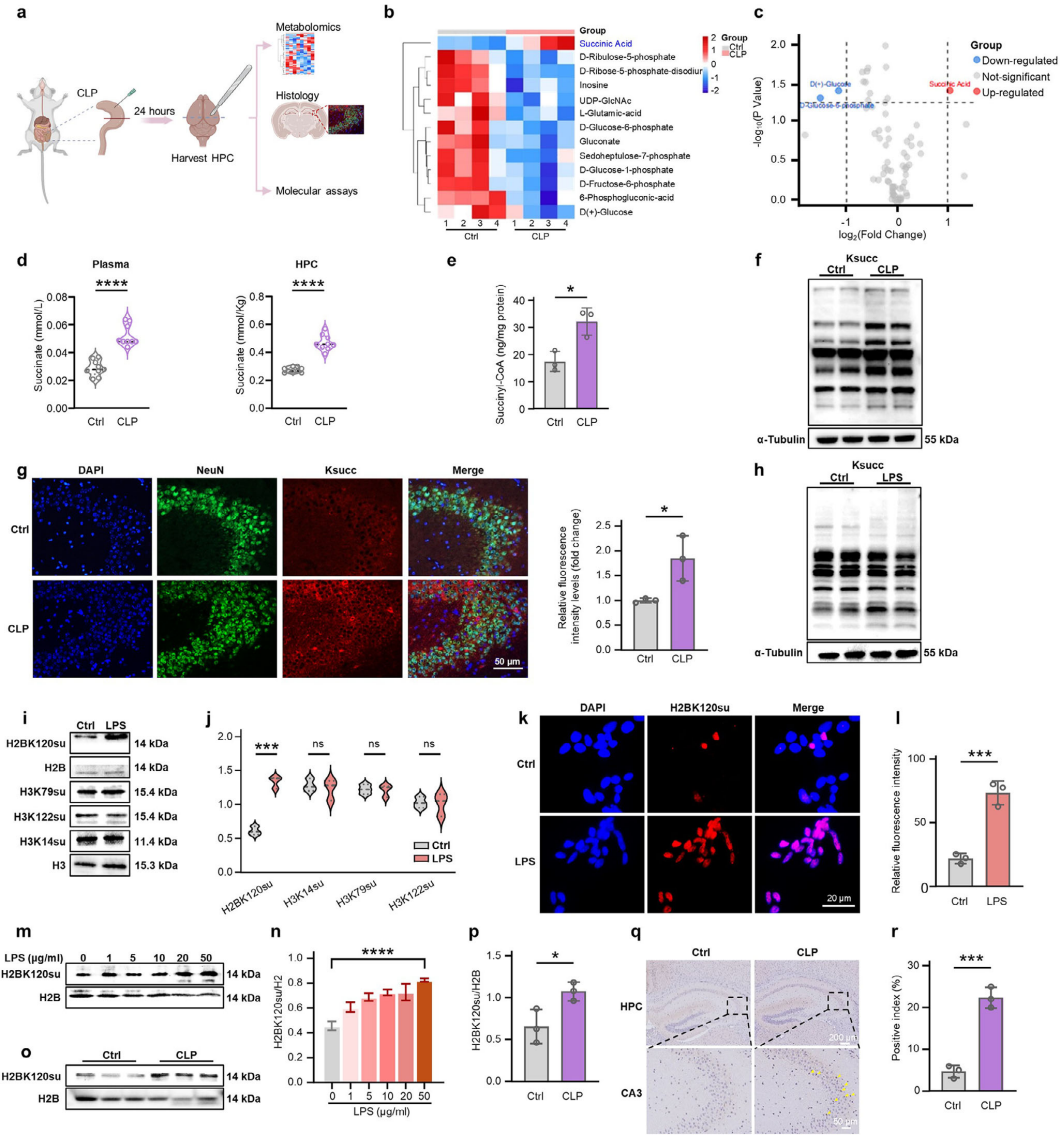
Increased histone H2BK120 succinylation in the hippocampus of CLP mice and LPS‐treated HT22 cells. (a) Experimental design for energy metabolism analysis in CLP mice. (b) Heatmap of targeted metabolomic profiling in hippocampal tissues from control and CLP mice. (c) Volcano plot showing differentially abundant metabolites in the hippocampus of control and CLP mice, n = 4 mice per group. (d) Plasma and hippocampal levels of succinate in control and CLP mice 24 h after surgery, n = 9 mice per group. (e) Hippocampal succinyl‐CoA levels in control and CLP mice 24 h after surgery, normalized to total protein content, n = 4 mice per group. (f) Western blot analysis of hippocampal Ksucc levels in CLP mice 24 h after surgery. Each lane represents one mouse, n = 6 mice per group. (g) Representative images and quantitative analysis of Ksucc co‐immunostaining with NeuN in hippocampal tissues in CLP mice 24 h after surgery. Scale bar, 50 µm, n = 3 mice per group. (h) Western blot analysis of Ksucc levels in HT22 cells after 24 h of LPS stimulation (50 µg/ml), n = 3 independent cell culture experiments. (i) Western blot analysis and quantitative results (j) of site‐specific histone succinylation in HT22 cells after 24 h of LPS stimulation, n = 3 independent cell culture experiments. (k) Representative images and quantitative analysis (l) of H2BK120su immunostaining in HT22 cells after 24 h of LPS stimulation. Scale bar, 20 µm, n = 3 independent cell culture experiments. (m) Western blot analysis and quantitative results (n) of H2BK120su levels in HT22 cells treated with different concentrations of LPS, n = 3 independent cell culture experiments. (o) Western blot analysis and quantitative results (p) of hippocampal H2BK120su levels in CLP mice 24 h after surgery. Each lane represents one mouse, n = 3 mice per group. (q) Representative immunohistochemical images and quantitative analysis (r) of H2BK120su in hippocampal tissues in CLP mice 24 h after surgery. Scale bar, 20 µm. Yellow arrows indicate H2BK120su‐positive cells, n = 3 mice per group. CLP, cecal ligation and puncture; LPS, lipopolysaccharide; Ksucc, lysine succinylation. Data are shown as mean ± SD. For animal experiments, n indicates the number of mice per group; for HT22 cell experiments, n indicates independent cell culture experiments. Two‐group comparisons were analyzed using unpaired two‐tailed Student's t‐test. Data in panel n were analyzed using one‐way ANOVA followed by Dunnett's post hoc test. ^*^ *p* < 0.05, ^**^ *p* < 0.01, ^***^ *p* < 0.001, and ^****^ *p* < 0.0001; ns, not significant.

Given the profound disruption of amino acid and nucleotide metabolism observed in SAE, which may impact TCA cycle anaplerosis, we next performed targeted metabolomics using LC‐MS/MS to quantify energy‐related metabolites. This analysis aimed to precisely evaluate alterations in the hippocampal energy metabolic axis in a murine model of sepsis (Figure 1a). The heatmap and volcano plot further showed a pronounced increase in succinate levels in the hippocampus of CLP mice, accompanied by significant decreases in glucose and glucose‐6‐phosphate (Figure 1b,c). Consistent with untargeted metabolomics, pathway enrichment analysis indicated that the differentially abundant metabolites were primarily associated with glycolysis and the tricarboxylic acid (TCA) cycle, suggesting substantial metabolic reprogramming in the septic hippocampus. Notably, the elevation of organic acids, particularly succinate, along with enhanced amino acid‐related metabolic signatures, suggested possible disturbance of succinate/succinyl‐CoA‐related metabolism, which may serve as substrates for protein succinylation and contribute to neuronal energetic stress and epigenetic dysregulation (Figure S2a). Additionally, biochemical assays revealed significantly elevated succinate levels in the serum and hippocampal tissue of CLP mice (Figure 1d). We further measured hippocampal succinyl‐CoA levels by ELISA and found a significant increase in CLP mice compared with controls (Figure 1e).

Given that succinyl‐CoA is the direct acyl donor for Ksucc, and that elevated succinate indicates disruption of the TCA cycle intermediate pool, particularly at the succinyl‐CoA/succinate node [14], we next assessed global protein succinylation in hippocampal tissue by immunoblotting and found global Ksucc was increased (Figure 1f). Histone succinylation was particularly elevated, and immunofluorescence analysis revealed strong Ksucc signals colocalizing with hippocampal neurons, with minimal signal detected in microglia or astrocytes (Figure 1g; Figure S2b,c), suggesting neuron‐specific succinylation changes in sepsis. To corroborate these findings in vitro, lipopolysaccharide (LPS) was applied to HT22 neuronal cells, resulting in increased Ksucc levels on immunoblots (Figure 1h) and elevated Ksucc signal intensity via immunofluorescence (Figure S2d,e). In addition, diethyl succinate (DES) further increased Ksucc levels in LPS‐stimulated neurons (Figure S2f). To identify specific succinylated histone residues, we examined multiple lysine sites and found that H2BK120 succinylation (H2BK120su) was significantly increased in LPS‐treated neurons (Figure 1I,j). Immunofluorescence staining further corroborated this observation (Figure 1k,l). This increase was dose‐dependent upon LPS stimulation (Figure 1m,n). Consistent with the in vitro findings, H2BK120su levels were significantly elevated in the hippocampus of CLP mice compared with sham‐operated controls, as shown by immunoblotting (Figure 1o,p) and immunohistochemistry (Figure 1q,r). Collectively, these results demonstrate that succinylation is upregulated in the hippocampus of CLP‐induced SAE mice, with a prominent and neuron‐specific increase in H2BK120su.

### 2.3 Pharmacological Inhibition of H2BK120 Succinylation Attenuates Neuronal Injury and Cognitive Impairment in SAE

**FIGURE 2:**
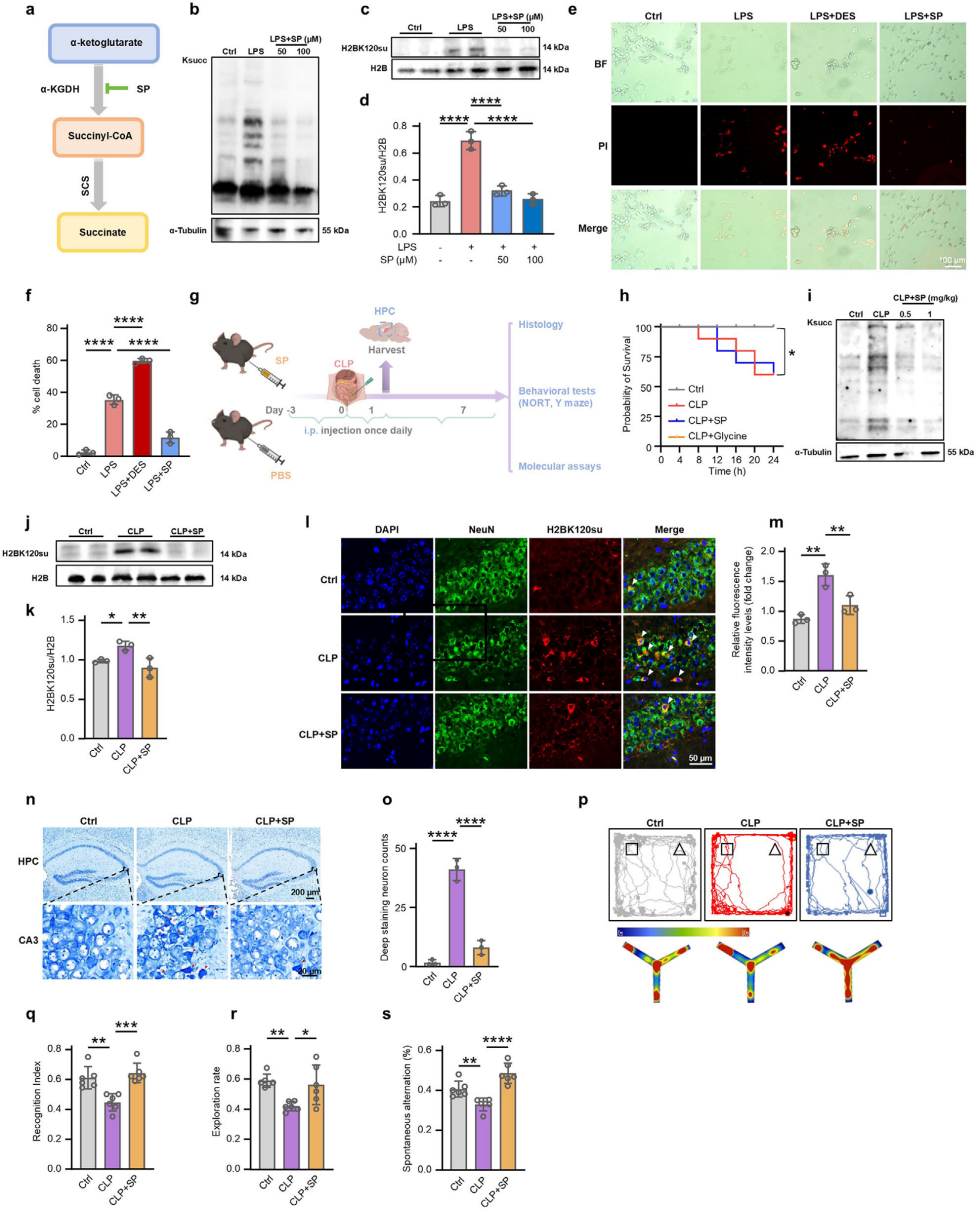
Inhibition of histone H2BK120 succinylation alleviates CLP‐induced sepsis‐associated encephalopathy. (a) Proposed schematic of the in vivo mechanisms through which SP reduces succinyl‐CoA levels. (b) Western blot analysis showing Ksucc and H2BK120su (c,d) levels in HT22 cells pretreated with SP (50 or 100 µM) for 3 h before LPS stimulation, n = 3 independent cell culture experiments. (e) Representative propidium iodide staining images and quantitative analysis (f) of HT22 cells pretreated with DES (10 mM) or SP (50 µM) for 3 h prior to LPS stimulation. PI enters cells with compromised plasma membranes and intercalates into double‐stranded DNA, emitting red fluorescence. Scale bar, 100 µm, n = 3 independent cell culture experiments. (g) Schematic illustration of the experimental design for SP administration in CLP mice. SP was intraperitoneally injected once daily for three consecutive days prior to CLP, with an additional injection administered on the day of surgery, to inhibit the generation of succinyl‐CoA. (h) Kaplan–Meier survival curves of SP‐pretreated mice within 24 h following CLP, n = 10 mice per group. (i) Western blot analysis showing hippocampal Ksucc and H2BK120su (j,k) levels in CLP mice pretreated with SP (0.5 mg/kg). Each lane represents one mouse, n = 3 mice per group.(l) Representative images and quantitative analysis (m) of H2BK120su co‐immunostaining with the neuronal marker NeuN in hippocampal tissues in CLP mice pretreated with SP. Scale bar, 50 µm, n = 3 mice per group. (n) Representative Nissl staining images and quantitative analysis (o) of hippocampal neurons in CLP mice pretreated with SP. Red arrows indicate Nissl bodies. In sham‐operated mice, hippocampal neurons exhibited normal morphology and organization, whereas CLP induced neuronal disorganization, vacuolization, and increased dark, shrunken Nissl bodies. Scale bars, 200 µm (top) and 20 µm (bottom), n = 3 mice per group. (p) Representative movement trajectories of Ctrl, CLP, and CLP + SP mice in the novel object recognition test (NORT), together with representative movement heatmaps showing exploratory distribution during the Y‐maze spontaneous alternation test, n = 6 mice per group. (q) Recognition index (RI) in the novel object recognition test among different experimental groups of mice, n = 6 mice per group. (r) Exploration ratio in the novel object recognition test among different experimental groups of mice, n = 6 mice per group. (s) Statistical analysis of spontaneous alternation percentage in the Y‐maze spontaneous alternation test in mice from different experimental groups, n = 6 mice per group. SP, succinyl‐phosphonate. DES, diethyl succinate. Data are shown as mean ± SD. For animal experiments, n indicates the number of mice per group; for HT22 cell experiments, n indicates independent cell culture experiments. Data in panel h were analyzed by the log‐rank test. Data in panels (d,f,k,m,o,q–s) were analyzed by one‐way ANOVA followed by Dunnett's multiple comparisons test. ^*^ *p* < 0.05, ^**^ *p* < 0.01, ^***^ *p* < 0.001, and ^****^ *p* < 0.0001; ns, not significant.

To investigate the contribution of succinate/succinyl‐CoA‐related metabolic disturbances to neuronal injury in sepsis, we performed metabolic interventions in both cellular and animal models. Based on prior evidence that succinyl‐phosphonate (SP), a selective inhibitor of the α‐ketoglutarate dehydrogenase complex (OGDH complex) in the TCA cycle, reduces succinyl‐CoA accumulation and subsequent protein succinylation (Figure 2a) [15, 16], we pretreated HT22 neuronal cells with SP before LPS stimulation. The results showed that both global Ksucc and H2BK120su were reduced (Figure 2b–d). Immunofluorescence confirmed that the elevated Ksucc induced by LPS was attenuated by pretreatment with SP (Figure S3a,b). Likewise, SP significantly diminished LPS‐induced H2BK120su fluorescence in HT22 cells (Figure S3c,d). To further complement the pharmacological inhibition data, we performed siRNA‐mediated knockdown of *Ogdh* in LPS‐treated HT22 cells. Succinyl‐CoA levels were measured by ELISA and normalized to total protein concentration. LPS increased intracellular succinyl‐CoA accumulation, whereas both SP treatment and *Ogdh* knockdown significantly reduced succinyl‐CoA levels under LPS stimulation (Figure S3e). Consistently, western blot analysis showed that *Ogdh* knockdown markedly attenuated LPS‐induced H2BK120su elevation (Figure S3f). We next examined whether altered succinylation affects neuronal survival. Propidium iodide (PI) staining revealed that LPS induced death in approximately 35% of HT22 cells, which was further increased by DES. SP pretreatment effectively rescued neuronal viability (Figure 2e,f). Similarly, *Ogdh* knockdown reduced the number of PI‐positive cells under LPS stimulation, indicating that genetic suppression of OGDHc‐related metabolic flux also alleviated LPS‐induced neuronal cell death (Figure S3g). Together, these findings indicate that OGDHc‐dependent succinyl‐CoA accumulation promotes LPS‐induced H2BK120 succinylation and neuronal injury, while both SP treatment and genetic *Ogdh* knockdown exert protective effects.

Subsequently, we evaluated whether reducing succinylation improves cognitive function in SAE mice. SP was administered intraperitoneally for 3 days before cecal ligation and puncture (CLP) (Figure 2g). Survival analysis showed that SP did not alter overall survival after CLP (Figure 2h). Moreover, besides global Ksucc, SP significantly decreased H2BK120su in hippocampal tissue (Figure 2i–k), consistent with immunofluorescence observations (Figure 2l,m). Nissl staining revealed that CLP induced severe neuronal damage, including vacuolization, disorganization, and dark shrunken nuclei, which was markedly alleviated by SP pretreatment (Figure 2n,o). Correspondingly, TUNEL staining indicated increased apoptosis in CLP mice, and this effect was attenuated by SP (Figure S3h,i). Cognitive performance was evaluated using the novel object recognition (NORT) and Y‐maze tests [17]. SP‐treated septic mice spent more time exploring the novel object and showed a higher recognition index than CLP controls (Figure 2p–r). In the Y‐maze, SP significantly restored the CLP‐induced decrease in spontaneous alternation percentage (Figure 2s). Collectively, these findings demonstrate that preoperative SP administration effectively reduces hippocampal neuronal H2BK120su, attenuates neuronal injury, and improves sepsis‐associated memory and cognitive deficits, thereby highlighting the therapeutic potential of targeting succinylation in SAE.

### 2.4 Protein Succinylation Triggers PINK1/Parkin‐Driven Mitophagy to Mediate Hippocampal Neuronal Injury in SAE

**FIGURE 3:**
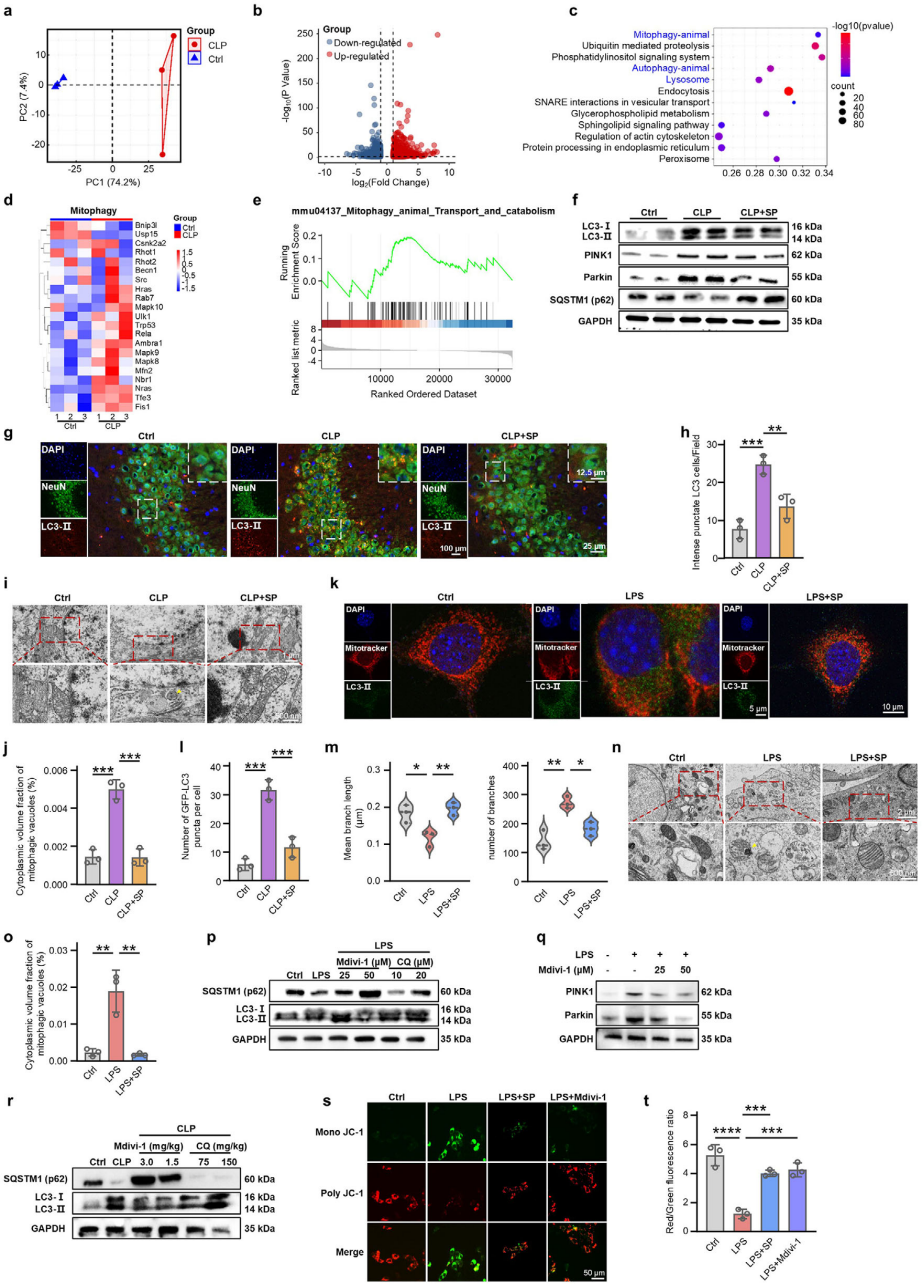
Succinylation triggers excessive PINK1‐dependent mitophagy in hippocampal neurons of CLP mice and in LPS‐stimulated HT22 cells. (a) PCA plot showing a clear separation between the sham and CLP groups, n = 3 mice per group. (b) Volcano plot showing upregulated and downregulated genes in the hippocampus after CLP. Differentially expressed genes (DEGs) were identified using univariate analysis (P < 0.05 and |log2FC| ≥ 1). (c) KEGG pathway enrichment analysis of alternative splicing (AS) events identified in the hippocampus of sham and CLP mice. (d) Heatmap showing alternatively spliced genes enriched in the mitophagy pathway in the hippocampus. (e) Gene Set Enrichment Analysis (GSEA) plot showing enrichment of the mitophagy pathway in the hippocampus. (f) Western blot analysis showing the expression of LC3‐II/I, PINK1, Parkin, SQSTM1 (p62), and TOM20 in hippocampal tissues from sham, CLP, and CLP + SP mice. Each lane represents one mouse, n = 3 mice per group. (g) Representative immunofluorescence images and quantitative analysis (h) of LC3‐II puncta in NeuN‐positive hippocampal neurons. Scale bars, 50 µm (left), 12.5 µm (upper right), and 25 µm (lower right), n = 3 mice per group. (i) Representative transmission electron microscopy (TEM) images and quantitative analysis (j) of mitophagosome‐like structures in hippocampal neurons. Yellow arrows mark mitochondria enclosed within autophagosomes. Scale bars, 1 µm (upper) and 500 nm (lower), n = 3 mice per group. (k) Representative confocal images and quantitative analysis (l) showing co‐localization of the mitochondrial‐specific fluorescent probe MitoTracker with the autophagy marker LC3‐II in HT22 cells. Scale bars, 5 µm (left) and 10 µm (right), n = 3 independent cell culture experiments. (m) Quantification of the alterations in mitochondrial morphology, including mean branch length and number of branches, based on multiple cells from three independent cell culture experiments. (n) Representative TEM images and quantitative analysis (o) of HT22 cells. Yellow arrows mark mitochondria enclosed within autophagosomes. Scale bars, 2 µm (upper) and 500 nm (lower), n = 3 independent cell culture experiments. (p) Western blot analysis showing LC3‐II/I and SQSTM1 (p62) expression in HT22 cells treated with Mdivi‐1 (25 or 50 µM) or CQ (10 or 20 µM) for 2 h following LPS stimulation, n = 3 independent cell culture experiments. (q) Western blot analysis showing PINK1 and Parkin expression in HT22 cells treated with Mdivi‐1 (25 or 50 µM) for 2 h following LPS stimulation, n = 3 independent cell culture experiments. (r) Western blot analysis showing LC3‐II/I and SQSTM1 (p62) expression in hippocampal tissues from mice pretreated with Mdivi‐1 (3.0 or 1.5 mg/kg) or CQ (75 or 150 mg/kg) 12 h prior to CLP and collected 24 h after surgery. Each lane represents one mouse, n = 3 mice per group. (s) Representative JC‐1 staining images and quantitative analysis (t) of LPS‐stimulated HT22 cells treated with SP (50 µM) or Mdivi‐1 (25 µM). The red/green fluorescence ratio indicates mitochondrial membrane potential. Scale bar, 50 µm, n = 3 independent cell culture experiments. Data are shown as mean ± SD. For animal experiments, n indicates the number of mice per group; for HT22 cell experiments, n indicates independent cell culture experiments unless otherwise stated. Data in panels (h,j,l–m,o, t) were analyzed by one‐way ANOVA followed by Dunnett's multiple comparisons test. ^*^ *p* < 0.05, ^**^ *p* < 0.01, ^***^ *p* < 0.001, and ^****^ *p* < 0.0001; ns, not significant.

Existing evidence indicates that mitophagy plays a critical role in neuroinflammation and neuronal apoptosis in SAE [18, 19, 20]. To delineate the complex pathological mechanisms affecting hippocampal neurons after sepsis, we performed transcriptome profiling of hippocampal 1 day after CLP. PCA revealed a clear separation between the CLP and control groups, indicating distinct global transcriptional profiles (Figure 3a). A total of 2257 differentially expressed genes (DEGs) were identified (1220 upregulated and 1037 downregulated; P < 0.05, |log2FC| ≥ 1), as visualized by volcano plot and heatmap (Figures 3b and S4a). Gene Ontology (GO) enrichment highlighted DEGs involvement in inflammatory response, extracellular matrix, and receptor‐ligand activity (Figure S4b). Kyoto Encyclopedia of Genes and Genomes (KEGG) pathway analysis further showed enrichment in IL‐17 signaling pathway, TNF signaling pathway, and cytokine‐cytokine receptor interaction (Figure S4c). Expanding beyond DEG‐based enrichment, we analyzed alternative splicing events and found significant splicing changes in pathways related to synaptic function and cellular homeostasis, including synaptic vesicle cycle, axon guidance, and mitophagy (Figure S4d). Further analysis revealed that ubiquitin‐mediated proteolysis, phosphatidylinositol signaling system, autophagy, and lysosome pathways were also enriched, suggesting coordinated regulation of mitophagy through splicing and membrane dynamics (Figure 3c). A heatmap of alternatively spliced genes in the mitophagy pathway showed condition‐dependent patterns (Figure 3d), and gene set enrichment analysis (GSEA) confirmed significant mitophagy pathway activation (Figure 3e).

We next assessed mitophagy activation at the protein level. Western blotting showed increased LC3‐II (a marker of autophagosome formation) and decreased SQSTM1/p62 and TOM20 in CLP hippocampal tissue, consistent with enhanced mitophagy (Figure S5a,b). Given the enrichment of ubiquitin‐mediated proteolysis in splicing analysis, we examined PINK1 and Parkin, key regulators of ubiquitin‐dependent mitophagy [21]. Both were upregulated after CLP, indicating engagement of the PINK1/Parkin pathway (Figure S5a,b). Notably, pretreatment with SP effectively mitigated the CLP‐associated increase in PINK1/Parkin signaling and attenuated the mitophagy‐related changes (Figures 3f and S5c). Immunofluorescence staining showed increased LC3‐II puncta in NeuN‐positive hippocampal neurons of CLP mice, suggesting enhanced autophagosome accumulation in neurons, which was attenuated by SP treatment (Figure 3g,h). Transmission electron microscopy (TEM) provided ultrastructural evidence of mitophagosomes in hippocampal neurons after CLP, with double‐membrane structures engulfing mitochondria; SP pretreatment reduced these features and improved mitochondrial morphology (Figure 3i,j). In LPS‐stimulated HT22 cells, we observed similar increases in LC3‐II, PINK1, and Parkin, accompanied by decreased p62 and TOM20 (Figure S5d,e). Immunofluorescence using MitoTracker and LC3‐II showed enhanced mitochondria‐autophagosome colocalization after LPS exposure, indicating active mitophagy (Figure 3k,l). Analysis of mitochondrial dynamics revealed that LPS shifted mitochondria toward a fragmented phenotype, as evidenced by shortened mean branch length and increased branch number, whereas SP treatment largely reversed these effects and preserved mitochondrial network integrity (Figure 3m). TEM of HT22 cells confirmed increased mitophagic structures under LPS stimulation, which was reversed by SP (Figure 3n,o). Together, these results indicate that CLP induces PINK1/Parkin‐dependent mitophagy in hippocampal neurons, and this response is attenuated by SP pretreatment.

To further determine whether LPS‐induced mitophagy reflects increased autophagic flux, we perturbed the pathway pharmacologically [22, 23]. In LPS‐stimulated HT22 cells, mitochondrial division inhibitor 1 (Mdivi‐1) increased p62 and reduced LC3‐II, PINK1, and Parkin (Figure 3p,q), while lysosomal inhibitor chloroquine (CQ) caused accumulation of both p62 and LC3‐II (Figure 3p). These patterns suggest that LPS‐induced LC3‐II accumulation reflects, at least in part, enhanced autophagic flux rather than a simple pre‐existing blockade of lysosomal degradation. Both Mdivi‐1 and CQ reduced LPS‐induced cell death, as measured by PI staining (Figure S5f,g). In CLP mice, Mdivi‐1 increased hippocampal p62 and decreased LC3‐II, supporting attenuation of excessive mitophagy‐related activation in vivo, whereas CQ administered 12 h before CLP showed no evident effect (Figure 3r). After optimizing the administration window, CQ given 18 h after CLP, particularly at 150 mg/kg, restored p62 expression and further increased LC3‐II accumulation (Figure S5h), indicating effective blockade of late‐stage autophagic degradation. These findings suggest that restraining mitophagy at different steps can partially rescue neuronal injury.

Subsequently, we further assessed mitochondrial dysfunction using JC‐1 probe for membrane potential and DCFH‐DA for ROS. LPS depolarized mitochondria and increased ROS in HT22 cells, while SP pretreatment reversed these effects (Figure 3s,t). Mdivi‐1 similarly protected mitochondrial function (Figures 3s,t and S5i,j). In CLP mice, Mdivi‐1 reduced TUNEL‐positive cells in the hippocampus, indicating attenuated neuronal apoptosis. Notably, optimized CQ treatment administered 6 h before tissue collection also decreased hippocampal TUNEL‐positive cells, suggesting that late‐stage autophagy blockade may alleviate neuronal apoptosis when applied within an appropriate treatment window (Figure S5k,l). Collectively, these results demonstrate that interventions targeting succinylation or limiting excessive mitochondrial fission can improve mitochondrial homeostasis and thereby partially rescue sepsis‐associated neuronal injury.

### 2.5 H2BK120 Succinylation Upregulates PD‐1 Expression and Triggers PD‐L1 Mitochondrial Translocation Leading to PINK1‐Dependent Mitophagy Activation

**FIGURE 4:**
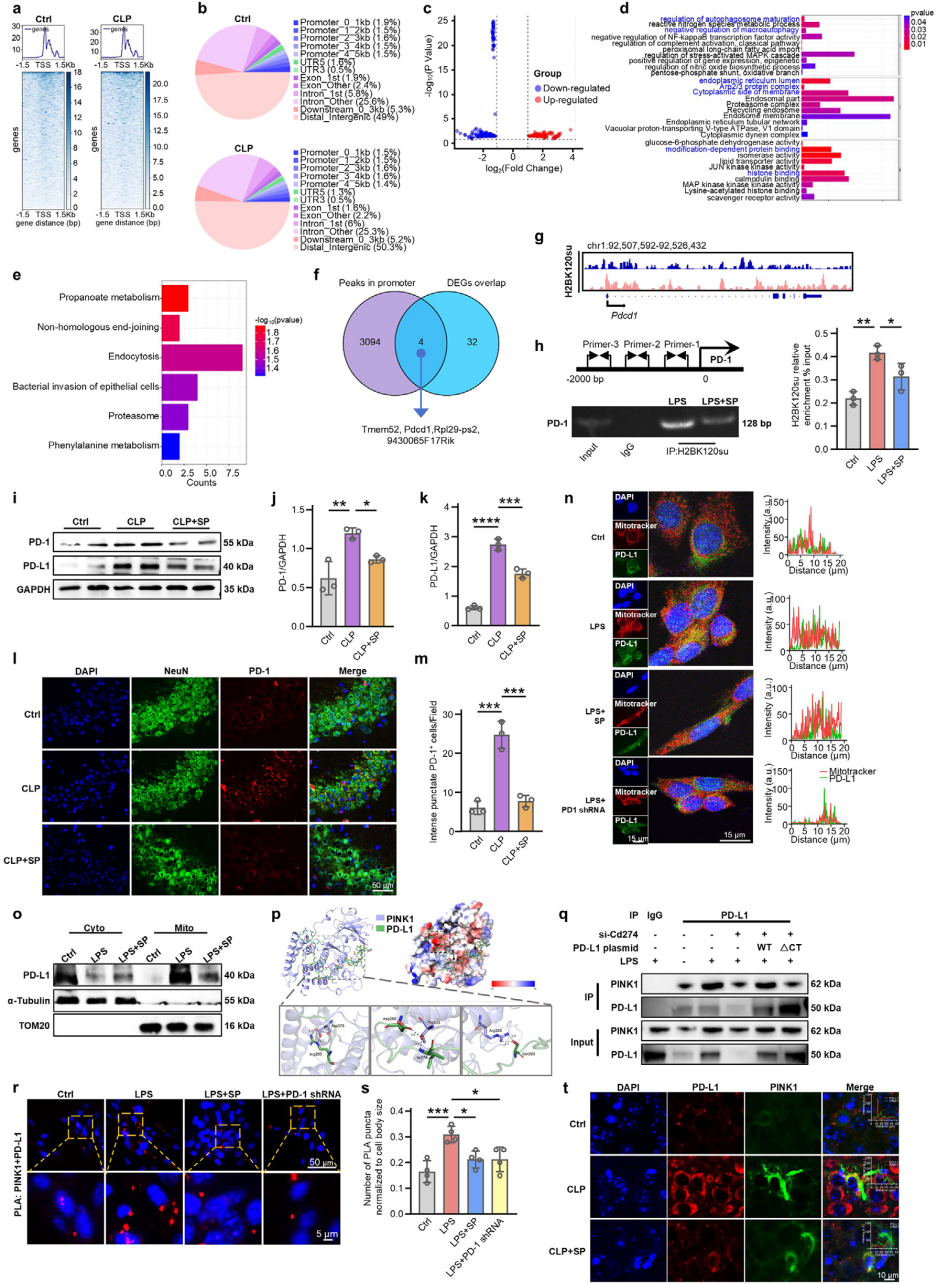
H2BK120 succinylation upregulates PD‐1 and promotes PD‐L1 mitochondrial translocation to activate PINK1‐dependent mitophagy.(a) Heatmap showing H2BK120su enrichment around transcription start sites (TSSs) in hippocampal CUT&Tag analysis. (b) Pie chart showing the distribution of differentially modified H2BK120su regions. (c) Volcano plot showing differentially enriched H2BK120su peaks. (d) GO pathway enrichment analysis of differentially H2BK120su‐modified genes. (e) KEGG pathway enrichment analysis of differentially H2BK120su‐modified genes. (f) Bioinformatic analysis identified Pdcd1 as a potential downstream target regulated by H2BK120su modification. (g) Integrated Genomics Viewer (IGV) tracks showing H2BK120su enrichment at the Pdcd1 locus in hippocampal CUT&Tag analysis. CUT&Tag was performed using hippocampal tissues from n = 2 mice per group. (h) Chromatin immunoprecipitation followed by qPCR (ChIP‐qPCR) analysis showing H2BK120su enrichment at the Pdcd1 promoter region, n = 3 independent cell culture experiments. (i) Western blot analysis and quantitative analysis (j‐k) showing PD‐1 and PD‐L1 protein expression in hippocampal tissues from sham, CLP, and CLP + SP mice. Each lane represents one mouse, n = 3 mice per group. (l) Representative immunofluorescence images and quantitative analysis (m) showing PD‐1 expression in NeuN‐positive neurons in the hippocampal CA3 region. Scale bar, 50 µm, n = 3 mice per group. (n) Representative confocal images and quantitative co‐localization analysis illustrating PD‐L1 and MitoTracker fluorescence overlap in HT22 cells. Scale bar, 15 µm, n = 3 independent cell culture experiments. (o) Western blot analysis showing PD‐L1 expression in the cytoplasmic (Cyto) and mitochondrial (Mito) fractions of HT22 cells, n = 3 independent cell culture experiments. (p) Molecular docking analysis revealed the putative binding interface between PD‐L1 and PINK1, predicting Asp375, Thr333, and Arg326 on PINK1 as key residues involved in the interaction. (q) Representative Co‐IP analysis of the interaction between PD‐L1 and PINK1 in HT22 cells. Endogenous *Cd274* was silenced, and cells were reconstituted with PD‐L1 WT or PD‐L1 ΔCT, followed by LPS stimulation. Cell lysates were immunoprecipitated with anti‐PD‐L1 or control IgG and immunoblotted with the indicated antibodies, n = 3 independent cell culture experiments.(r) Representative proximity ligation assay (PLA) images showing PINK1‐PD‐L1 interactions in neuronal cell bodies in different groups. Scale bars, 50 µm (left) and 5 µm (right), n = 3 independent cell culture experiments. (s) Quantitative analysis of PLA puncta per neuronal soma following the indicated treatments, normalized to cell body size, n = 3 independent cell culture experiments. (t) Representative immunofluorescence images and quantitative co‐localization analysis showing co‐localization of PD‐L1 and PINK1 in the hippocampal CA3 region. Scale bar, 10 µm, n = 3 mice per group. Data are shown as mean ± SD. For animal experiments, n indicates the number of mice per group; for HT22 cell experiments, n indicates independent cell culture experiments. Data in panels (h,j–k,m,s) were analyzed by one‐way ANOVA followed by Dunnett's multiple comparisons test. ^*^ *p* < 0.05, ^**^ *p* < 0.01, ^***^ *p* < 0.001, and ^****^p < 0.0001; ns, not significant.

Histone modifications can influence transcription of target genes [24]. To investigate the role of histone succinylation in regulating gene expression in SAE, we performed CUT&Tag profiling of H2BK120su in hippocampal tissue from CLP mice. Approximately 8% of H2BK120su‐enriched peaks overlapped promoter regions, with the majority distributed in intergenic regions (Figure 4a,b). Differential analysis identified 27 056 significantly elevated and 26 933 decreased H2BK120su peaks in CLP mice (Figure 4c). GO enrichment analysis of genes associated with differential H2BK120su peaks revealed terms related to autophagosome maturation, macroautophagy regulation, endoplasmic reticulum lumen, Arp2/3 complex, and modification‐dependent protein binding, suggesting a link between H2BK120su and autophagy‐cytoskeletal remodeling processes (Figure 4d). KEGG pathway analysis further indicated enrichment in propanoate metabolism, endocytosis, proteasome, and phenylalanine metabolism, implicating H2BK120su in metabolic and protein turnover in the septic hippocampus (Figure 4e).

Integration of CUT&Tag data with hippocampal transcriptomic profiling from CLP mice identified *Pdcd1* (encoding PD‐1) and *Tmem52* as candidate genes regulated by H2BK120su (Figure 4f). Focused analysis on Pdcd1 revealed strong H2BK120su enrichment at its promoter region in CLP mice (Figure 4g). Chromatin immunoprecipitation‐quantitative PCR (ChIP‐qPCR) further confirmed specific H2BK120su occupancy at multiple sites within the Pdcd1 promoter (Figure 4h). A *Pdcd1* promoter luciferase assay further showed that H2B‐WT enhanced LPS‐induced promoter activity, whereas H2B‐K120R largely abolished this effect (Figure S6a), supporting a functional role of H2B K120 in *Pdcd1* transcriptional activation. Concordantly, *Pdcd1* and *Tmem52* mRNA levels were elevated in CLP hippocampal tissue, along with increased expression of *Cd274*, which encodes the PD‐1 ligand PD‐L1 (Figure S6b). Immunoblotting confirmed increased PD‐1 and PD‐L1 protein levels in the hippocampus after CLP, an effect reversed by SP pretreatment (Figures S6c,d and 4i–k). Immunofluorescence showed enhanced colocalization of PD‐1 and PD‐L1 with hippocampal neurons in CLP mice, which was attenuated by SP (Figures 4l,m and S6e,f). Similarly, LPS‐stimulated HT22 cells exhibited increased PD‐1 and PD‐L1 expression (Figure S6g,h).

Existing evidence indicates that PINK1‐mediated mitochondrial distribution of PD‐L1 contributes to autophagy‐dependent PD‐L1 degradation [25]. We next examined PD‐L1 subcellular localization. In LPS‐treated HT22 cells, PD‐L1 showed increased colocalization with mitochondria, an effect reduced by SP pretreatment or Pdcd1 knockdown (Figures 4n and S6i). To further characterize PD‐L1 redistribution, we isolated cytosolic and mitochondrial fractions and confirmed LPS‐induced translocation of PD‐L1 to mitochondrial, which was largely abolished by SP pretreatment (Figure 4o). Molecular docking suggested that the cytoplasmic tail of PD‐L1 binds the kinase domain of PINK1 via electrostatic interactions involving residues Arg265, Asp268/Ile274, and Ser283 (Figure 4p). Consistently, deletion of the PD‐L1 cytoplasmic tail impaired LPS‐induced mitochondrial localization of PD‐L1 and weakened its interaction with PINK1 in *Cd274*‐silenced cells reconstituted with PD‐L1 WT or PD‐L1 ΔCT (Figures 4q and S6j). Co‐immunoprecipitation (Co‐IP) and proximity ligation assays demonstrated a physical interaction between PINK1 and PD‐L1 that was enhanced by LPS and diminished by SP or Pdcd1 silencing (Figures 4r,s and S6k,l). Immunofluorescence in hippocampal tissue corroborated increased PINK1 and PD‐L1 colocalization in CLP mice, which was mitigated by SP (Figure 4t).

Furthermore, functional studies showed that neutralizing PD‐1 or PD‐L1 antibodies, or Pdcd1 knockdown, reduced LC3‐II levels, increased p62, and diminished mitochondria‐LC3 colocalization, indicating attenuated mitophagy (Figure S6m–p). These interventions also decreased LPS‐induced neuronal death (Figure S6q,r). Collectively, these findings demonstrate that sepsis‐induced H2BK120su drives transcriptional upregulation of PD‐1 at the *Pdcd1* promoter, facilitating PD‐L1 translocation to mitochondria and interacting with PINK1, which promotes excessive mitophagy and neuronal injury. Intervention with SP attenuates this pathological cascade by suppressing H2BK120su and PD‐1/PD‐L1 activation.

### 2.6 SIRT7 Downregulation Mediates H2BK120su Accumulation and Neuronal Injury in SAE, which is Rescued by SIRT7 Activation

**FIGURE 5:**
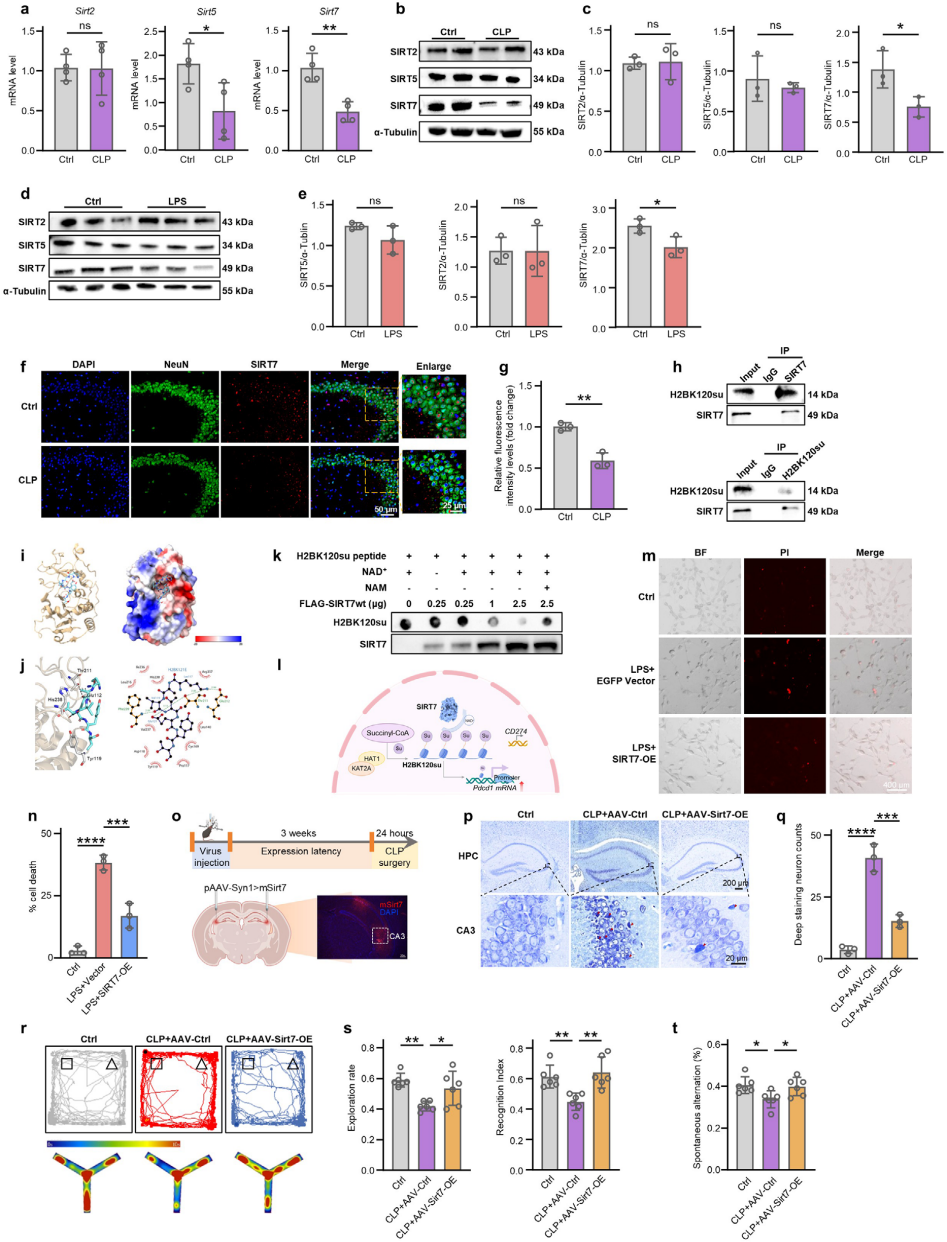
Overexpression of hippocampal neuronal SIRT7 effectively mitigates CLP‐induced neuronal injury in sepsis‐associated encephalopathy. (a) qRT‐PCR analysis showing the mRNA expression levels of *Sirt2*, *Sirt5* and *Sirt7* in hippocampal tissues, n = 3 mice per group. (b) Western blot and quantitative analysis (c) showing the protein expression levels of SIRT2, SIRT5, and SIRT7 in hippocampal tissues. Each lane represents one mouse, n = 3 mice per group. (d) Western blot and quantitative analysis (e) showing the protein expression levels of SIRT2, SIRT5, and SIRT7 in HT22 cells, n = 3 independent cell culture experiments. (f) Representative immunofluorescence images and quantitative analysis (g) showing SIRT7 expression in NeuN‐positive neurons in hippocampal CA3 region. Scale bars, 50 µm (left) and 25 µm (right), n = 3 mice per group. (h) Co‐IP analysis showing a physical interaction between H2BK120su and SIRT7 in HT22 cells, n = 3 independent cell culture experiments. (i) Overall molecular docking structure of the SIRT7 (gold)‐H2BK120su peptide (gray) complex (left) and electrostatic surface potential of SIRT7 at the peptide‐binding interface (right). (j) Surface molecular docking model of the SIRT7 (gold)‐H2BK120su peptide (gray) complex (left), and LIGPLOT representation illustrating key interactions between the H2BK120su peptide and SIRT7 (right). The H2B peptide fragment (purple) and key residues of SIRT7 (brown) are shown in ball‐and‐stick representation. Carbon atoms are shown in black, nitrogen atoms in blue, oxygen atoms in red, and magenta dashed lines indicate hydrogen bonds mediating direct interactions. (k) Recombinant SIRT7 was incubated with H2BK120su peptide (1 µg) in the presence or absence of NAD^+^ (1.0 mM) and NAM (10 mM). H2BK120su levels were detected by dot blot using an anti‐H2BK120su antibody, and SIRT7 input was verified by Western blotting, n = 3 independent in vitro reactions. (l) Schematic illustration of the mechanism underlying increased H2BK120 succinylation. (m) Representative propidium iodide staining images and quantitative analysis (n) showing cell death in HT22 cells after SIRT7 overexpression. Scale bar, 400 µm, n = 3 independent cell culture experiments. (o) Schematic diagram illustrating stereotaxic viral injection into the hippocampal CA3 region. (p) Representative images and quantitative analysis (q) of Nissl staining in mouse hippocampal neurons, with Nissl bodies indicated by red arrows. Scale bars, 200 µm (top) and 20 µm (bottom), n = 3 mice per group. (r) Representative movement trajectories of Ctrl, CLP + AAV‐Ctrl, and CLP + AAV‐Sirt7‐OE mice in the NORT, together with representative movement heatmaps showing exploratory distribution during the Y‐maze spontaneous alternation test, n = 6 mice per group. (s) Quantification of the recognition index in the novel object recognition test and the exploration rate in mice from different experimental groups, n = 6 mice per group. (t) Spontaneous alternation percentage in the Y‐maze spontaneous alternation test, n = 6 mice per group. Data are shown as mean ± SD. For animal experiments, n indicates the number of mice per group; for HT22 cell experiments, n indicates independent cell culture experiments. Two‐group comparisons were analyzed using unpaired two‐tailed Student's t‐test. For panels (n,q,s,t) data were analyzed using one‐way ANOVA followed by Dunnett's post hoc test. ^*^ *p* < 0.05, ^**^ *p* < 0.01, ^***^ *p* < 0.001 and ^****^ *p* < 0.0001; ns, not significant.

We next investigated the regulatory enzymes governing Ksucc in the septic hippocampus, given the pronounced increase in histone H2BK120su. Ksucc is dynamically regulated by specific writer enzymes, such as HAT1, CBP/p300, and KAT2A that add succinyl groups, and eraser enzymes, including SIRT5, SIRT7, and HDAC1/2/3 that remove them. Additionally, enzymes involved in succinyl‐CoA metabolism, such as DLST, FH1, SUCLG1/2, SUCLA2, and CPT1A, influence substrate availability for succinylation [9, 10]. qRT‐PCR analysis revealed downregulation of the desuccinylases *Sirt5* and *Sirt7*, as well as *Hdac1*, alongside upregulation of the acetyltransferases *Hat1* and *Kat2a* and the succinyl‐CoA synthetase subunits *Suclg1* and *Suclg2* in CLP hippocampus (Figures 5a and S7a). Immunoblotting confirmed a marked reduction in SIRT7 protein abundance (Figure 5b,c), suggesting that elevated hippocampal succinylation in sepsis may result from both enhanced writer activity and impaired eraser function, particularly reduced SIRT7. In LPS‐stimulated HT22 cells, SIRT7 protein levels also decreased, but not SIRT5 or SIRT2 (Figure 5d,e), and immunofluorescence analysis showed reduced SIRT7 colocalization with NeuN^+^ hippocampal neurons in CLP mice, as well as decreased SIRT7 fluorescence in HT22 cells following LPS treatment (Figures 5f,g and S7b). Co‐IP confirmed a physical interaction between SIRT7 and H2BK120su (Figure 5h).

To elucidate the structural basis of this interaction, we performed molecular docking between SIRT7 and an H2B peptide (residues 117–123) carrying H2BK120su under an NAD^+^‐free condition. The docking model revealed that the peptide binds an elongated, negatively charged groove in SIRT7, stabilized by hydrogen bonds (such as Thr211 with H2B Glu121; Glu212 with H2B Lys117; Phe239 with H2B Val119) and hydrophobic interactions involving Leu216, Ile236, and others (Figure 5i,j). In vitro deacylation assays further showed that recombinant SIRT7 reduced H2BK120su peptide signals in a NAD^+^‐dependent and nicotinamide‐sensitive manner (Figure 5k). Moreover, Co‐IP analysis of Flag‐H2B‐WT and Flag‐H2B‐K120R revealed that K120R mutation diminished H2BK120su signals and attenuated the association between H2B and SIRT7(Figure S7c). These data support a mechanism whereby SIRT7 downregulation impairs nuclear desuccinylation, leading to H2BK120su accumulation (Figure 5l). Altered expression of succinyl‐CoA synthetase (SCS) subunits (*Suclg1*, *Suclg2*, *Sucla2*) suggested metabolic contributions to succinyl‐CoA availability [26]. Overexpression of individual SCS subunits modulated global Ksucc levels (Figure S7d), indicating that shifts in SCS‐mediated flux influence succinylation by altering substrate supply. Together, these results highlight SIRT7 as a key regulator of H2BK120su dynamics under metabolic stress.

On this basis, we subsequently focused on the nuclear desuccinylase SIRT7 to define its role in the dynamic regulation of H2BK120su and thereby influencing neuronal viability. To assess the functional impact of SIRT7, we first overexpressed it in HT22 cells and observed that SIRT7 upregulation significantly attenuated LPS‐induced cell death (Figures 5m,n and S7e). In vivo, neuron‐specific AAV‐mediated overexpression of SIRT7 in the CA3 hippocampus did not affect overall survival (Figures 5o and S7f–h) but reduced neuronal damage, as shown by improved Nissl staining (Figure 5p,q), and rescued cognitive deficits in novel object recognition and Y‐maze tests (Figure 5r–t). These findings demonstrate that selective SIRT7 upregulation preserves neuronal integrity and mitigates sepsis‐associated cognitive impairment, underscoring its therapeutic potential in SAE.

### 2.7 Neuron‐Specific SIRT7 Deletion Exacerbates Hippocampal Injury and Cognitive Dysfunction in SAE by Promoting H2BK120su and Mitophagy

**FIGURE 6:**
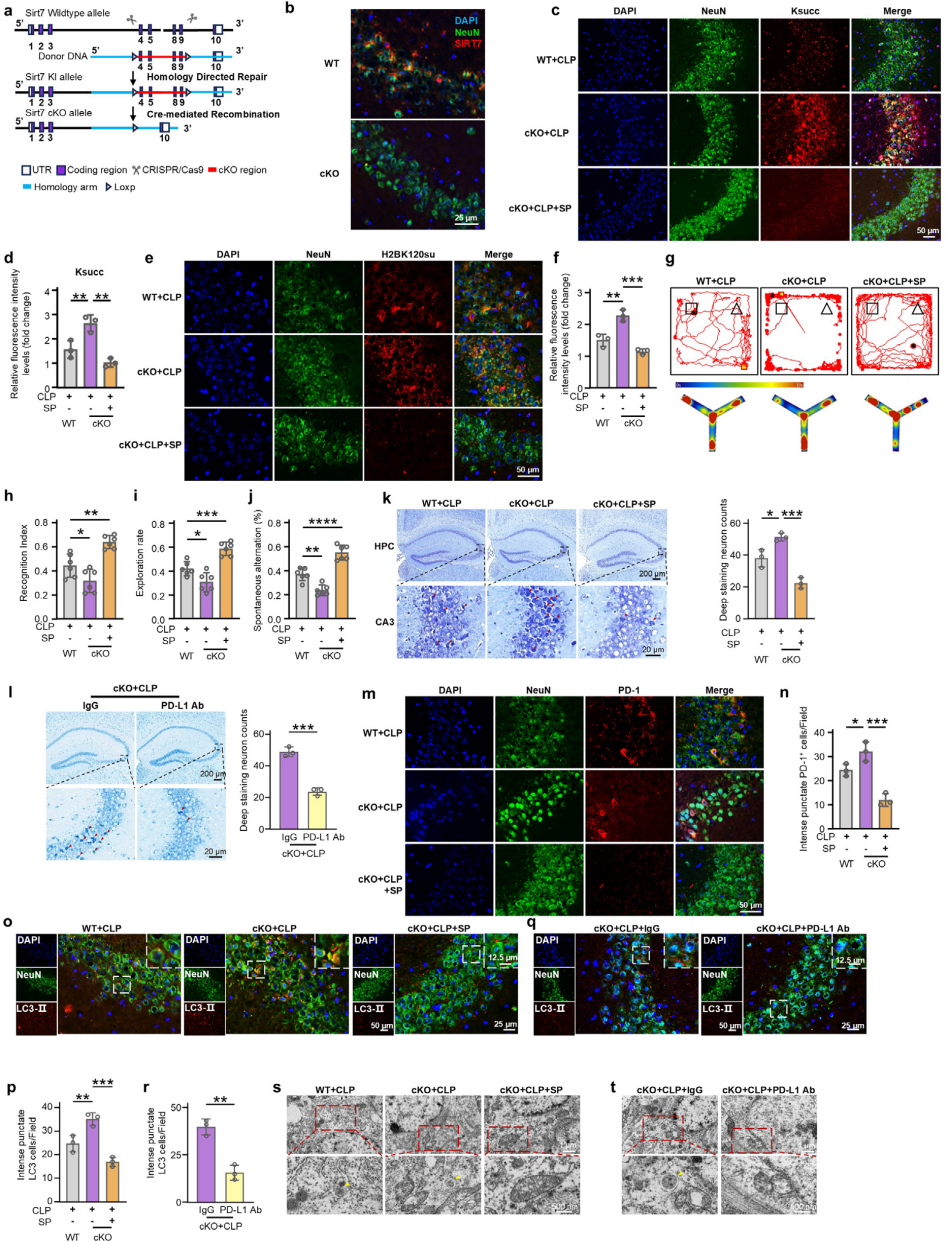
Neuron‐specific deletion of SIRT7 markedly aggravated cognitive dysfunction in mice subjected to sepsis. (a) Schematic diagram illustrating the strategy for neuron‐specific deletion of SIRT7. (b) Representative immunofluorescence images showing SIRT7 expression in NeuN‐positive hippocampal neurons of WT and SIRT7‐cKO mice. Scale bar, 50 µm, n = 3 mice per group. (c) Representative immunofluorescence images and quantitative analysis (d) showing Ksucc expression in NeuN‐positive hippocampal neurons of WT + CLP, cKO + CLP, and cKO + CLP + SP mice. Scale bar, 50 µm, n = 3 mice per group. (e) Representative immunofluorescence images and quantitative analysis (f) showing H2BK120su expression in NeuN‐positive hippocampal neurons of WT + CLP, cKO + CLP, and cKO + CLP + SP mice. Scale bar, 50 µm, n = 3 mice per group. (g) Representative movement trajectories of WT + CLP, cKO + CLP, and cKO + CLP + SP mice in the novel object recognition test, together with representative movement heatmaps showing exploratory distribution during the Y‐maze spontaneous alternation test, n = 6 mice per group. (h) Quantification of the recognition index in the NORT and the exploration rate (i) in mice from different experimental groups. (j) Statistical analysis of spontaneous alternation percentage in the Y‐maze spontaneous alternation test in mice from different experimental groups. (k) Representative images and quantitative analysis of Nissl staining of WT + CLP, cKO + CLP, and cKO + CLP + SP mice in the hippocampal CA3 region. Red arrows indicate Nissl bodies. Scale bars, 200 µm (top) and 20 µm (bottom), n = 3 mice per group. (l) Representative images and quantitative analysis of Nissl staining in hippocampal neurons from cKO + CLP mice treated with intra‐HPC IgG or anti‐PD‐L1 antibody. Red arrows indicate Nissl bodies. Scale bars, 200 µm (top) and 20 µm (bottom), n = 3 mice per group. (m) Representative immunofluorescence images and quantitative analysis (n) showing PD‐1 expression in NeuN‐positive hippocampal neurons of WT + CLP, cKO + CLP, and cKO + CLP + SP mice. Scale bar, 50 µm, n = 3 mice per group. (o) Representative confocal immunofluorescence images and quantitative analysis (p) illustrating the autophagy marker LC3‐II expression in NeuN‐positive hippocampal neurons of WT + CLP, cKO + CLP, and cKO + CLP + SP mice. Scale bars, 50 µm (left), 12.5 µm (upper right), and 25 µm (lower right), n = 3 mice per group. (q) Representative confocal immunofluorescence images and quantitative analysis (r) illustrating the autophagy marker LC3‐II expression in NeuN‐positive hippocampal neurons of cKO + CLP mice treated with intra‐HPC IgG or anti‐PD‐L1 antibody. Scale bars, 50 µm (left), 12.5 µm (upper right), and 25 µm (lower right), n = 3 mice per group. (s) Representative TEM images of hippocampal neurons of WT + CLP, cKO + CLP, and cKO + CLP + SP mice. Yellow arrows mark mitochondria enclosed within autophagosomes. Scale bars, 1 µm (top) and 500 nm (bottom), n = 3 mice per group. (t) Representative TEM images of hippocampal neurons of cKO + CLP mice treated with intra‐HPC IgG or anti‐PD‐L1 antibody. Yellow arrows mark mitochondria enclosed within autophagosomes. Scale bars, 1 µm (top) and 500 nm (bottom), n = 3 mice per group. Data are shown as mean ± SD. Data in l and r were analyzed using unpaired two‐tailed Student's t‐test. Data in (d,f,h–k,n,p) were analyzed by one‐way ANOVA followed by Dunnett's multiple comparisons test. ^*^ *p* < 0.05, ^**^ *p* < 0.01, ^***^ *p* < 0.001, and ^****^ *p* < 0.0001; ns, not significant.

Given the marked reduction of SIRT7 expression in hippocampal neurons after CLP, we generated a neuron‐specific SIRT7 conditional knockout mouse line (*Sirt7^fl/fl; Pkd2l1‐2A‐CreERT2*) to further investigate its role in SAE related cognitive impairment. Immunofluorescence confirmed efficient SIRT7 depletion in hippocampal neurons (Figure 6a,b). Although SIRT7 is well known as an NAD^+^‐dependent deacetylase, recent evidence also supports its role as a histone desuccinylase, particularly in the context of DNA damage response and cellular stress [27]. Immunoblotting revealed a marked increase in both global Ksucc and site‐specific H2BK120su in the hippocampus of SIRT7‐cKO septic mice. Both of these modifications were effectively abolished by SP intervention, whereas global Kac was unchanged, highlighting a succinylation‐specific effect (Figure S8a), immunofluorescence corroborated these findings (Figure 6c–f), whereas neither global Kac nor H2BK120ac showed appreciable differences (Figure S8b). Collectively, these results identify SIRT7 as a major nuclear desuccinylase regulating H2BK120su levels in the septic hippocampus.

To assess the functional impact of SIRT7 depletion, we evaluated neuronal integrity and cognitive performance. Behavioral tests revealed that SIRT7‐cKO septic mice exhibited significant cognitive deficits compared with CLP controls. Specifically, these mice showed reduced novel object preference and recognition index in the NORT, as well as impaired spontaneous alternation in the Y‐maze. Importantly, pharmacological inhibition of succinylation by SP largely rescued these deficits (Figure 6g–j). Nissl staining showed more severe hippocampal neuronal pathology in SIRT7‐cKO septic mice, including disorganized cellular architecture and increased dark, shrunken Nissl bodies. SP treatment and PD‐L1 neutralization both alleviated these morphological abnormalities, suggesting partial protection against hippocampal neuronal damage (Figure 6k,l). Furthermore, TUNEL staining showed that SP treatment significantly reduced hippocampal neuronal apoptosis, indicating that inhibition of succinylation attenuates SIRT7 deficiency‐aggravated neuronal injury during sepsis (Figure S8c,d). Immunofluorescence staining showed that PD‐1 and PD‐L1 expression in hippocampal neurons was further elevated in SIRT7‐cKO septic mice compared with CLP mice, whereas SP treatment markedly alleviated this increase (Figures 6m,n and S8e,f). Meanwhile, the colocalization between PD‐L1 and PINK1 was correspondingly altered (Figure S8g).

To determine whether SIRT7 deficiency aggravates sepsis‐induced mitophagy and neuronal ultrastructural injury, we performed immunofluorescence and TEM analysis. Immunofluorescence showed increased LC3‐II autophagosomes in NeuN^+^ hippocampal neurons in SIRT7‐cKO septic mice, which was reduced by SP pretreatment or PD‐L1 blockade (Figure 6o–r). Ultrastructural examination by TEM confirmed more double‐membrane‐enclosed damaged mitochondria in hippocampal neurons of SIRT7‐cKO septic mice, alongside widened synaptic clefts and thinning of postsynaptic densities. These pathological changes were significantly ameliorated by SP pretreatment or PD‐L1 blockade (Figures 6s,t and S8h–m). Together, these findings demonstrate that CA3 neuron‐specific deletion of SIRT7 heightens susceptibility to sepsis‐driven excessive mitophagy, synaptic disruption, and cognitive impairment, whereas suppressing succinylation restores neuronal homeostasis.

### 2.8 SIRT7‐Mediated Histone Succinylation Promotes Excessive Mitophagy via the PD‐1/PD‐L1 Axis

**FIGURE 7:**
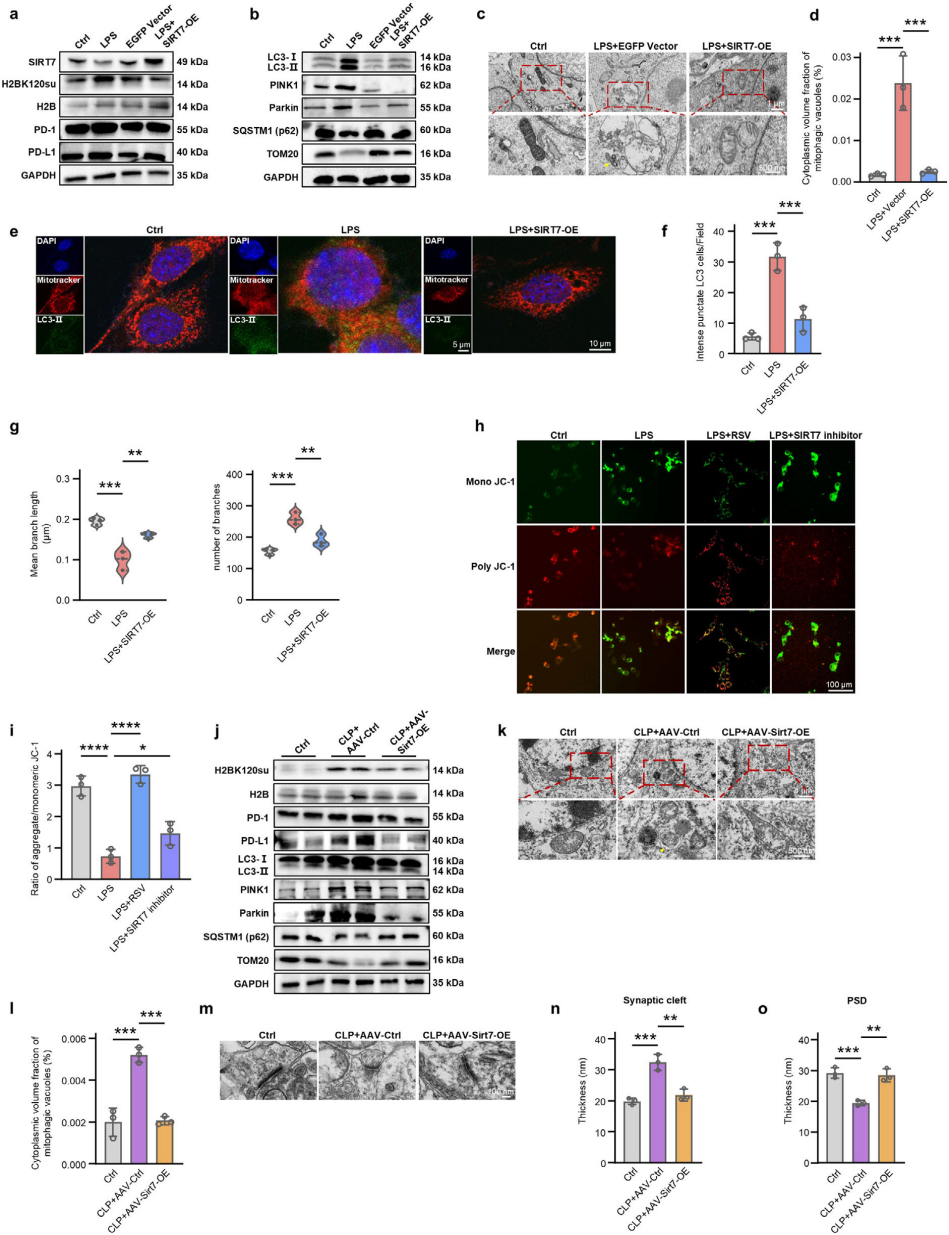
SIRT7‐mediated histone succinylation promotes excessive autophagy activation via the PD‐1/PD‐L1 axis. (a) Western blot analysis showing the expression levels of SIRT7, H2BK120su, PD‐1, and PD‐L1 in HT22 cells after SIRT7 overexpression, n = 3 independent experiments. (b) Western blot analysis showing the protein expression levels of LC3‐II/I, PINK1, Parkin, SQSTM1 (p62), and TOM20 in HT22 cells after SIRT7 overexpression, n = 3 independent experiments. (c) Representative TEM images and quantitative analysis (d) of HT22 cells. Yellow arrows mark mitochondria enclosed within autophagosomes. Scale bars, 1 µm (top) and 500 nm (bottom), n = 3 independent experiments. (e) Representative confocal images and quantitative analysis (f) showing co‐localization of the mitochondrial‐specific fluorescent probe MitoTracker with the autophagy marker LC3‐II in HT22 cells. Scale bars, 5 µm (left) and 10 µm (right), n = 3 independent experiments. (g) Quantification of the alterations in mitochondrial morphology, including mean branch length and number of branches, n = 3 independent experiments. (h) Representative fluorescence images and quantitative analysis (i) of JC‐1 staining in HT22 cells following pretreatment with resveratrol (RSV) or a SIRT7 inhibitor. Scale bar, 100 µm, n = 3 independent experiments. (j) Western blot analysis showing the protein expression levels of H2BK120su, PD‐1, PD‐L1, and mitophagy‐related markers (LC3‐II/I, PINK1, Parkin, SQSTM1/p62, and TOM20) in the hippocampus after CA3 injection of AAV‐Sirt7‐OE, n = 3 mice per group. (k) Representative TEM images and quantitative analysis (l) illustrating mitophagy in hippocampal neurons after CA3 injection of AAV‐Sirt7‐OE. Yellow arrows mark mitochondria enclosed within autophagosomes. Scale bars, 1 µm (top) and 500 nm (bottom), n = 3 mice per group. (m) Representative TEM images illustrating synaptic ultrastructure. Scale bar, 100 nm, n = 3 mice per group. (n) Quantitative analysis of synaptic cleft width. (o) Quantitative analysis of postsynaptic membrane thickness. Data are shown as mean ± SD. Data in (d,f,g,i,l,n–o) were analyzed by one‐way ANOVA followed by Dunnett's multiple comparisons test. ^*^ *p* < 0.05, ^**^ *p* < 0.01, ^***^ *p* < 0.001, and ^****^ *p* < 0.0001; ns, not significant.

In addition to its role in maintaining nuclear genome integrity [28], SIRT7 has also been implicated in mitophagy regulation through deacetylation of Parkin in septic acute kidney injury [29]. However, whether SIRT7 regulates mitophagy via histone succinylation in SAE remains unknown. Here, we overexpressed SIRT7 in HT22 cells. Immunoblot analysis showed that SIRT7 upregulation reduced H2BK120su and downregulated PD‐1 and PD‐L1 protein levels, suggesting that SIRT7 suppresses PD‐1/PD‐L1 axis activation, at least in part, through histone succinylation (Figures 7a and S9a). Furthermore, relative to LPS alone, SIRT7 overexpression decreased LC3‐II accumulation, reduced PINK1 and Parkin expression, and increased p62 and TOM20 levels, indicating that SIRT7 attenuates mitophagy activation via the PINK1/Parkin pathway (Figures 7b and S9b). Accompanying these changes, LPS‐impaired ATP production was restored upon SIRT7 upregulation (Figure S9c). Consistently, TEM imaging showed a reduction in mitophagosome formation (Figure 7c,d), and increased colocalization of LC3‐II with MitoTracker further supported attenuated mitophagy (Figure 7e,f). Concomitantly, SIRT7 overexpression effectively attenuated mitochondrial fragmentation, preserving mitochondrial network integrity (Figure 7g).

Next, we evaluated mitochondrial function following SIRT7 modulation. Using the JC‐1 probe, we observed that SIRT7 overexpression restored the red/green fluorescence ratio in LPS‐challenged HT22 cells, indicating recovery of mitochondrial membrane potential (Figure S9d,e). Concurrently, DCFH‐DA staining showed a reduction in intracellular ROS in SIRT7‐overexpressing cells (Figure S9f,g). Pharmacological interventions further confirmed these effects: pretreatment with the SIRT7 activator resveratrol (RSV) enhanced the JC‐1 ratio and suppressed ROS production, whereas SIRT7 inhibitor treatment exacerbated mitochondrial depolarization and ROS accumulation (Figures 7h,i and S10a,b). Consistent with these findings, TUNEL staining confirmed that RSV reduced neuronal apoptosis, while SIRT7 inhibition increased cell death (Figure S10c,d). These results collectively suggest that SIRT7 downregulation promotes H2BK120su‐mediated PD‐1/PD‐L1 axis activation and hypermitophagy, leading to mitochondrial dysfunction and hippocampal neuronal injury.

Finally, we validated these findings in vivo by performing neuron‐specific SIRT7 overexpression in the hippocampal CA3 region of mice via AAV delivery. Following CLP‐induced sepsis, SIRT7‐overexpressing mice showed reduced H2BK120su levels and decreased PD‐1/PD‐L1 expression. Concurrently, LC3‐II, PINK1, and Parkin levels were diminished, while p62 and TOM20 were increased (Figures 7j and S9h). TEM analysis confirmed fewer mitophagosomes in hippocampal neurons (Figure 7k,l), and ultrastructural evaluation revealed narrower synaptic clefts and restored postsynaptic density thickness (Figure 7m–o). Together, these results indicate that SIRT7 overexpression attenuates sepsis‐induced hippocampal injury by suppressing H2BK120su‐PD‐1/PD‐L1 signaling and excessive mitophagy, thereby preserving synaptic integrity and mitigating cognitive deficits.

## 3 Discussion

SAE significantly contributes to sepsis‐related mortality and long‐term cognitive disability. While metabolic and inflammatory disturbances are recognized drivers of SAE, the role of post‐translational modifications, particularly those linking metabolism to epigenetic regulation, remains poorly defined. Our study demonstrated that sepsis‐induced metabolic reprogramming led to hippocampal succinate accumulation and consequently increased histone succinylation. We identified a specific epigenetic‐metabolic circuit wherein downregulation of the nuclear desuccinylase SIRT7 in neurons elevated histone H2BK120 succinylation (H2BK120su), which in turn promoted the transcription of the immune checkpoint molecule PD‐1. Concurrently, PD‐L1 translocated to mitochondria, interacted with PINK1, and drove excessive PINK1/Parkin‐dependent mitophagy. This cascade resulted in mitochondrial dysfunction, oxidative stress, and neuronal death, underpinning cognitive deficits in SAE. Interventions targeting this axis, either by inhibiting succinylation or augmenting SIRT7 activity, effectively mitigated neuronal injury and restored cognitive function.

Recent advances highlight metabolites as key mediators of epigenetic signaling. TCA cycle intermediates, such as succinyl‐CoA, serve as acyl donors for various lysine modifications, including succinylation, directly coupling cellular metabolic state to protein function and gene expression [30, 31, 32]. Consistent with this paradigm, our targeted metabolomics revealed a marked accumulation of succinate in the septic hippocampus, accompanied by a global increase in protein lysine succinylation (Ksuc). This likely reflects inflammation/hypoxia‐driven perturbations in mitochondrial TCA cycle flux and electron transport chain activity, leading to an altered succinyl‐CoA/succinate balance. The concurrent sepsis‐associated depletion of NAD^+^ may further impair sirtuin‐mediated desuccinylation, exacerbating succinylome remodeling. Notably, we found that this metabolic shift manifests prominently in a site‐specific histone modification—H2BK120su. The increase in H2BK120su, observed both in vivo and in LPS‐stimulated neurons, was attenuated by SP, an OGDHc inhibitor that limits succinyl‐CoA generation. Because SP pharmacologically inhibits OGDHc and may broadly affect TCA‐cycle flux, the SP‐based findings should be interpreted together with genetic validation rather than as evidence from pharmacological inhibition alone. Consistent with the effects of SP, siRNA‐mediated *Ogdh* knockdown reduced succinyl‐CoA accumulation, attenuated H2BK120su elevation, and decreased LPS‐induced cell death in HT22 cells, supporting the involvement of OGDHc‐dependent metabolic disturbance in neuronal succinylation and injury. These findings suggest the pathophysiological relevance of histone succinylation in SAE.

Protein succinylation is dynamically regulated by “writer” and “eraser” enzymes [13, 33]. In the septic hippocampus, we observed a molecular profile that promotes increased succinylation. This was characterized by the upregulation of potential “writer” enzymes (such as HAT1 and KAT2A) and succinyl‐CoA synthetase subunits, together with the downregulation of key “eraser” enzymes—most notably SIRT7 at the protein level. Notably, *Sirt7* mRNA expression was also significantly decreased in the hippocampus of CLP‐induced septic mice compared with control mice, suggesting that transcriptional repression may at least partially contribute to SIRT7 downregulation in SAE. Although SIRT5 is widely recognized as a major mitochondrial desuccinylase, its protein levels remained unchanged in both the hippocampus of CLP‐induced septic mice and LPS‐treated HT22 neurons in our model, suggesting that SIRT5 may not be a primary driver of the observed succinylation alterations under septic conditions. In contrast, as a nuclear histone desuccinylase, SIRT7 can directly regulate histone succinylation and participate in the DNA damage response and cell survival. Neuron‐specific Sirt7 knockout exacerbated sepsis‐induced H2BK120su, neuronal injury, and cognitive impairment, whereas neuronal SIRT7 overexpression or activation conferred substantial protection. Together, these findings establish SIRT7 as a primary regulator of hippocampal H2BK120su in sepsis and a key mediator of neuronal homeostasis. Nevertheless, although the reduction in *Sirt7* mRNA supports a transcriptional component, our current data do not fully exclude the involvement of additional mechanisms, such as enhanced protein degradation or post‐translational modifications affecting SIRT7 stability or activity, which warrants further investigation.

Our CUT&Tag and transcriptomic analyses identified *Pdcd1*, which encodes PD‐1, as a candidate downstream gene associated with H2BK120su enrichment. Sepsis‐induced H2BK120su enrichment at the Pdcd1 promoter correlated with increased PD‐1 expression in hippocampal neurons. This expands the role of the PD‐1/PD‐L1 axis beyond immunology into direct neuronal pathophysiology. While PD‐L1 expression may be co‐regulated by inflammatory signals (e.g., JAK‐STAT1‐IRF1), its subcellular localization was dramatically altered by sepsis [31, 32, 33, 34, 35]. We found that PD‐L1 translocated from the plasma membrane to mitochondria in neurons, where it physically interacted with PINK1, as validated by co‐IP, PLA, and molecular docking. This redistribution was dependent on the succinylation‐PD‐1 axis, as it was reversed by SP or Pdcd1 knockdown. Notably, studies in tumor cells have proposed that an ATAD3A‐PINK1 axis promotes PD‐L1 redistribution to mitochondrial compartments and have suggested that PINK1 can interact with the cytoplasmic tail of PD‐L1 [25, 36]. These observations provide a relevant precedent for the concept that membrane protein trafficking routes can be rewired under stress and coupled to mitochondrial stress signaling [24]. Together, our findings establish a novel link between an epigenetic‐metabolic signal (H2BK120su), a surface receptor axis (PD‐1/PD‐L1), and a core mitochondrial quality control apparatus (PINK1). Nevertheless, although H2BK120su enrichment at the *Pdcd1* promoter, ChIP‐qPCR validation, and promoter reporter assays support a regulatory link between H2BK120su and *Pdcd1* transcription, our current data do not fully distinguish whether H2BK120su directly activates *Pdcd1* transcription or indirectly facilitates transcription through broader chromatin remodeling, altered nucleosome accessibility, or recruitment of additional transcriptional regulators. Therefore, H2BK120su should be interpreted as a regulatory epigenetic mark associated with *Pdcd1* activation rather than definitive proof of direct transcriptional activation by this single modification.

The functional consequence of this convergence was the excessive activation of PINK1/Parkin‐dependent mitophagy. Sepsis led to robust upregulation of PINK1/Parkin, increased LC3‐II, and depletion of p62 and TOM20 in hippocampal neurons, with ultrastructural evidence further confirming enhanced mitophagic activity. Pharmacological and genetic evidence suggests that the H2BK120su‐PD‐1/PD‐L1 axis is positioned upstream of this process. In particular, neuron‐specific *Sirt7* knockout provided genetic evidence linking SIRT7 loss to enhanced H2BK120su and mitophagy activation, whereas the downstream involvement of PD‐1/PD‐L1 was supported by antibody neutralization and knockdown‐based approaches. Blocking PD‐1/PD‐L1 or reducing H2BK120su attenuated mitophagy markers and mitochondrial dysfunction (loss of ΔΨm, ROS overproduction). Notably, inhibiting mitophagy at different nodes—either upstream via SP (targeting succinylation) or Mdivi‐1 (inhibiting fission), or downstream via PD‐1/PD‐L1 blockade—improved neuronal survival. We recognize that basal mitophagy is generally neuroprotective, as it removes damaged mitochondria and preserves mitochondrial quality control in neurons [37, 38]. Therefore, our findings should be interpreted as a state of “mitophagy overload” rather than mitophagy activation per se. In our SAE model, sustained PINK1/Parkin activation was accompanied by TOM20 loss, mitochondrial dysfunction, abundant mitophagosomes, and neuronal injury, while partial suppression of this pathway improved neuronal survival. This pattern differs from “failed mitophagy,” in which impaired initiation or incomplete autophagic flux leads to the accumulation of damaged mitochondria [39, 40]. Thus, the pathogenic threshold likely reflects a functional imbalance between mitochondrial damage, mitophagic clearance, and mitochondrial renewal, rather than a single quantitative marker. This indicates that sepsis‐triggered metabolic‐epigenetic signals drive mitophagy beyond homeostatic regulatory capacity, transforming it from a protective mechanism into a driver of neuronal energy crisis and death.

Notably, although PD‐L1 neutralization alleviated neuronal injury and reduced the abundance of mitophagosomes, ultrastructural analyses revealed persistent mitochondrial damage, including membrane rupture and vacuolization. These observations suggest that while neuronal PD‐1/PD‐L1 blockade effectively suppresses excessive mitophagy and promotes neuronal survival, it is insufficient to fully restore mitochondrial structural integrity, highlighting the need for therapeutic strategies that simultaneously target neuron‐intrinsic PD‐1/PD‐L1 signaling and directly protect mitochondrial homeostasis under septic stress. Although we observed mitochondrial dysfunction, including loss of ΔΨm and ROS overproduction, the present study did not directly assess mitochondrial respiratory activity, such as oxygen consumption rate or respiratory chain complex function. Therefore, whether mitochondrial PD‐L1 directly affects neuronal mitochondrial respiration independently of mitophagy remains to be further investigated. Nevertheless, we acknowledge that the causal requirement of PD‐L1 in PINK1/Parkin‐dependent mitophagy has not yet been validated using PD‐L1‐deficient or neuron‐specific PD‐L1 loss‐of‐function models. Future studies employing global or neuron‐specific Cd274/PD‐L1 knockout mice, or conditional neuronal PD‐L1 deletion, would further clarify whether neuronal PD‐L1 is indispensable for SAE‐related mitophagy activation and neuronal injury. Several additional limitations should also be acknowledged. First, only male mice were used in the present study; therefore, potential sex‐dependent differences in SIRT7 expression, histone succinylation, PD‐1/PD‐L1 signaling, and mitophagy activation during SAE remain unknown. Second, our findings have not yet been validated in human SAE samples. Future studies using human postmortem brain tissues, cerebrospinal fluid or blood‐based biomarkers, and human‐derived neuronal models will be important to determine whether the SIRT7‐H2BK120su‐PD‐1/PD‐L1‐PINK1 axis is conserved in patients with sepsis‐associated cognitive impairment.

In summary, our study underscores SIRT7 as a critical intracellular mediator in the progression of SAE by regulating histone succinylation. Activation of the SIRT7‐H2BK120su‐PD‐1/PD‐L1‐PINK1 axis contributes to excessive mitophagy mobilization, culminating in neuronal death. Accordingly, neuron‐targeted overexpression of SIRT7 or inhibition of H2BK120su attenuated sepsis‐induced neuronal loss and improved cognitive and memory deficits in mice. These findings suggest that therapeutically targeting the SIRT7‐H2BK120su pathway represents a promising strategy for treating sepsis‐associated cognitive impairment.

## 4 Experimental Section

### 4.1 Animals

Male C57BL/6N mice (6–8 weeks old, weighing 18–22 g) were purchased from Shanghai Yishang Biotechnology Co., Ltd. Sirt7^^^flox/flox^^^; Pkd2l1‐2A‐CreERT2^^+/−^^ mice were generated by MouseOne Biotechnology (Wuhan, China). All animal experiments were conducted in accordance with the Guidelines for the Care and Use of Laboratory Animals and approved by the Animal Ethics Committee of Shanghai First People's Hospital (No. 2023AW062).

The cecal ligation and puncture (CLP) model was established as previously described. Briefly, mice were anesthetized by intraperitoneal injection of 1% sodium pentobarbital (1 mg/kg), followed by a 1–2 cm midline abdominal incision. The cecum was carefully exposed, ligated with a 5‐0 silk suture at approximately 15 mm from the cecal tip, and punctured once with a 20‐gauge needle at the distal end. A small amount of fecal content was gently extruded, after which the cecum was returned to the abdominal cavity, and the abdominal wall was sutured. Sham‐operated mice underwent identical surgical procedures without cecal ligation or puncture. At the end of surgery, all mice received an intraperitoneal injection of 0.9% saline for fluid resuscitation and were allowed free access to food and water. Samples were collected at the indicated time points and either fixed in 4% paraformaldehyde or immediately stored at −80°C for further analysis.

### 4.2 In Vivo Treatment

To investigate the role of protein lysine succinylation in sepsis‐induced brain injury, mice received intraperitoneal injections of succinyl phosphonate trisodium salt (0.5 mg/kg body weight; MedChemExpress, HY‐12688A) once daily for three consecutive days prior to CLP or sham surgery, with an additional injection administered on the day of surgery, to inhibit the generation of succinyl‐CoA.

To inhibit mitochondrial fission or block autophagic flux, mice were intraperitoneally injected with Mdivi‐1 (1.5 or 3.0 mg/kg body weight; Selleck, S7162) or chloroquine (75 or 150 mg/kg body weight; Selleck, S6999) 12 h prior to CLP or sham surgery, followed by an additional injection immediately before surgery. For the optimized CQ treatment experiment, CQ (75 or 150 mg/kg body weight) was administered intraperitoneally 18 h after CLP surgery, and hippocampal tissues were collected 6 h later, corresponding to 24 h after CLP.

To block the central hippocampal PD‐1/PD‐L1 signaling axis, mice received bilateral intra‐hippocampal injection of a PD‐L1‐neutralizing antibody (2 µg/side; Selleck, A2115) 3 h prior to CLP. The antibody was diluted in sterile phosphate‐buffered saline. Control mice received an equal volume of isotype‐matched IgG control antibody via the same bilateral intra‐hippocampal injection procedure.

### 4.3 Cell Culture and Treatments

The HT22 mouse hippocampal neuronal cell line was purchased from Wuhan Servicebio Biotechnology Co., Ltd. Cells were cultured in DMEM/F‐12 medium (Gibco) supplemented with 5% fetal bovine serum and maintained in a humidified incubator at 37°C with 5% CO_2_. Unless otherwise indicated, HT22 cells were stimulated with lipopolysaccharide (LPS; Sigma, L2880) at 10 µg/mL for 24 h. For dose‐response experiments, HT22 cells were treated with LPS at 0, 1, 5, 10, 20, or 50 µg/mL for 24 h.

In separate experiments, HT22 cells were treated with succinyl phosphonate trisodium salt (50 or 100 µM), succinate diethyl ester (DES; 10 or 20 mM; Sigma, W237712), Mdivi‐1 (25 or 50 µM), chloroquine (10 or 20 µM), PD‐1 antibody (10 µg/mL; Selleck, A2122), PD‐L1 antibody (10 µg/mL; Selleck, A2115), resveratrol (RSV; 1 µM; MedChemExpress, HY‐16561), or the SIRT7 inhibitor 97491 (1 µM; MedChemExpress, HY‐135899), as indicated.

For RNA interference experiments, HT22 cells were transfected with siRNAs targeting mouse *Cd274* or *Ogdh*, or with shRNAs targeting mouse *Sirt7*, using Lipofectamine RNAiMAX or the corresponding plasmid transfection reagent according to the manufacturer's instructions. A negative control siRNA or empty vector was used as the corresponding control. The shRNA and siRNA sequences are listed in Table S1. Knockdown efficiency was verified by western blotting or qRT‐PCR before subsequent experiments.

For PD‐L1 rescue experiments, HT22 cells were transfected with mouse PD‐L1‐WT or PD‐L1‐ΔCT expression plasmids in the pcDNA3.1(+) backbone after endogenous *Cd274* knockdown. The PD‐L1‐ΔCT construct retained amino acids 1–260 and lacked the cytoplasmic tail. Plasmid information is listed in Table S2.

### 4.4 LC/MS Untargeted Metabolomics Analysis

Following sample pretreatment, untargeted metabolomic analysis was performed using a UHPLC system (Nexera X2 LC‐30AD, Shimadzu, Japan) coupled with a Q Exactive Plus mass spectrometer (Thermo Fisher Scientific, USA) at Shanghai Bioprofile Technology Company, Ltd. Metabolites were separated on an ACQUITY UPLC HSS T3 column (2.1 × 100 mm, 1.8 µm; Waters, Milford, MA, USA) with a mobile phase of 0.1% formic acid in water (A) and acetonitrile (B) under a gradient flow of 0.3 mL/min. Data were acquired in both positive and negative ion modes using a heated electrospray ionization source (HESI). Raw data were processed with MS‐DIAL for peak alignment and metabolite identification against HMDB, MassBank, and an in‐house spectral library. The processed data were further analyzed using SIMCA‐P (version 14.1, Umetrics, Umea, Sweden) for multivariate statistical analysis, including Pareto‐scaled principal component analysis (PCA) and partial least squares‐discriminant analysis (PLS‐DA).

### 4.5 Targeted Metabolomics

To profile energy metabolism‐related metabolites in the hippocampus, targeted metabolomics analysis was performed using hippocampal tissues from sham‐operated (n = 4) and CLP‐treated (n = 4) mice. A total of 80 energy metabolites were quantitatively detected. Targeted metabolomics detection and subsequent data analysis were conducted by Cosmos Wisdom Biotechnology Co., Ltd. (Hangzhou, China).

### 4.6 Measurement of Succinyl‐CoA Levels

Mouse hippocampal tissues or HT22 cells were lysed on ice, and the lysates were centrifuged at 12 000 × g for 10 min at 4°C. The supernatants were collected for subsequent analysis. Succinyl‐CoA levels were determined using a succinyl‐CoA ELISA kit (MM‐45785M1; MEIMIAN) according to the manufacturer's protocol. Absorbance was measured at 450 nm with a microplate reader, and sample concentrations were calculated based on the standard curve. Total protein concentrations of the same samples were measured using a BCA protein assay. Succinyl‐CoA content was normalized to total protein concentration and expressed as ng/mg protein.

### 4.7 Measurement of Succinate Levels

At 24 h after CLP, mouse serum and brain tissues were collected for succinate measurement. Succinate levels were determined using a commercial colorimetric assay kit (Succinate Acid Colorimetric Assay Kit, Elabscience, Cat. No. E‐BC‐K902‐M) according to the manufacturer's instructions. Briefly, serum samples were analyzed directly, whereas brain tissues were homogenized and centrifuged, and the supernatants were used for detection. Absorbance was measured at 555 nm with a microplate reader, and succinate concentrations were calculated from the standard curve. Serum levels were expressed as mmol/L, and brain tissue levels were normalized to wet tissue weight.

### 4.8 RNA Sequencing

Total RNA was extracted from hippocampal samples with the Universal RNA Extraction CZ Kit (RNC643, ONREW) according to the manufacturer's instructions. RNA quantity was analyzed using Qubit 4.0 (Invitrogen) and quality examined by electrophoresis on a denaturing agarose gel. RNA libraries were prepared using the VVAHTS Universal V8 RNA‐seq Library Prep Kit for Illumina (NR605‐0, Vazyme), followed sequencing using the Illumina NovaSeq 6000 platform with the 150 paired‐end sequencing strategy. Enrichment of mRNA, library construction, sequencing and data analysis were performed by Shanghai Xu Ran Biotechnology Co., Ltd.

The raw data was handled by Skewer v0.2.2 and data quality was checked by FastQC v0.11.2. The read length was 2 × 150 bp. clean reads were aligned to the mouse genome mm10 from ensembl using STAR, with one mismatch allowed. StringTie (v1.3.1c) was used to generate gene expression data, and differential gene expression was analyzed by DESeq2 (v1.16.1). The thresholds for determining DEGs are P < 0.05 and absolute fold change ≥ 2. Then DEGs were chosen for function and signaling pathway enrichment analysis using TopGO and KEGG database. The significantly enriched pathways were determined when P < 0.05.

### 4.9 CUT&TAG Analysis of H2BK120su Changes

The sequencing was conducted on the Illumina Novaseq X Plus platform (Shanghai Xuran Biotechnology Co., Ltd.). The raw data was quality‐assessed using Fastqc (v0.11.5) and Skewer (v0.2.2), and quality control analysis and statistics of the high‐quality base proportion were performed on the preprocessed data. The preprocessed sequences were compared with the reference genome sequence of mm10 using the bowtie2 software, and Peak Calling was performed using Macs2 (v2.2.9.1). Through homer (V4.11.2), the top 100 Peak position information with the highest confidence ranking was selected to predict potential Motifs.

Differential expression analysis is made using DEseq2, Data whose absolute value of log2Foldchange larger than 1 and pvalue less than 0.05 is kept for subsequent analysis. Input samples are used for removal of background noise during peak calls.

### 4.10 Lentiviral Transduction

Lentiviral vectors expressing *mSirt7* (VB250113‐1079frw) or shRNA targeting *mPdcd1* (VB250917‐2090hbg) were constructed and packaged by VectorBuilder (Guangzhou, China) (Table S2). HT22 cells were infected with the lentiviruses at a multiplicity of infection (MOI) of 100 in the presence of HiTransG reagent (GeneChem, Shanghai, China). Stable cell lines were selected with puromycin (2 µg/mL; Sigma‐Aldrich) after 48 h of infection, and transduction efficiency was verified by EGFP fluorescence and Western blotting.

### 4.11 Stereotaxic Intrahippocampal Microinjection

Stereotaxic adeno‐associated virus (AAV) injections into the hippocampal CA3 region were performed as previously described. Briefly, mice were anesthetized with 1.5%–2% isoflurane and positioned in a stereotaxic frame (RWD Life Science Co.). Ophthalmic ointment was applied throughout the procedure to prevent corneal drying. After a midline scalp incision and removal of overlying muscle at the designated coordinates, a cranial window of approximately 0.25 mm^2^ was drilled using a dental microdrill. An AAV vector expressing mouse Sirt7 under the control of the synapsin I (SYN1) promoter (AAV‐SYN1‐Sirt7‐P2A‐miRFP720) was used for neuronal overexpression in vivo. The virus was delivered bilaterally into the hippocampal CA3 region using a pulled glass micropipette (Model P‐97, Sutter Instrument; tip diameter, 10–20 µm) at an infusion rate of 20–25 nL/min. Approximately 100 nL of viral suspension (1 × 10^1^ ^2^ genome copies/µL) was injected per site. The stereotaxic coordinates for CA3 injection were as follows (relative to bregma): anteroposterior (AP), −2.5 mm; mediolateral (ML), ±2.5 mm; and dorsoventral (DV), −2.25 mm. After each injection, the micropipette was left in place for approximately 5 min and then slowly withdrawn to prevent reflux of the viral solution. Finally, the scalp was sutured, and mice were returned to their home cages for postoperative recovery.

### 4.12 Immunofluorescence (IF) Staining

For tissue immunofluorescence, hippocampal samples were collected 24 h after CLP or sham surgery, fixed in 4% paraformaldehyde (PFA), cryoprotected in 30% sucrose solution, embedded in optimal cutting temperature (OCT) compound, and sectioned at a thickness of 10 µm using a cryostat. Antigen retrieval was not required for frozen sections.

For cell immunofluorescence, cultured cells were washed three times with PBS and fixed in 4% PFA. Cell membranes were permeabilized with 0.1% Triton X‐100, and nonspecific binding was blocked with 1% bovine serum albumin (BSA) for 1 h at room temperature. The sections or cells were then incubated with primary antibodies diluted in 1% BSA at 4°C overnight. After washing, samples were incubated with fluorescence‐conjugated secondary antibodies (Cell Signaling Technology) for 1 h at room temperature in the dark. Finally, nuclei were counterstained with DAPI, and fluorescence images were captured using an Olympus fluorescence microscope.

### 4.13 Western Blot

We lysed cells using cell lysis buffer (NCM Biotech, WB3100) supplemented with protease inhibitor buffer (ShareBio, SB‐WB026). The BCA assay (NCM Biotech, WB6501) determined protein concentration. SDS‐PAGE separated equal amounts of extracts. The proteins were then transferred onto a polyvinylidene fluoride (PVDF) membrane (Millipore), which was blocked by 5% non‐fat milk (dissolved in Tris‐buffered saline (pH 7.4) containing 0.1% Tween‐20). The membranes were incubated with specific primary antibodies overnight, washed the next day, and incubated with horseradish peroxidase (HRP) ‐labeled secondary antibodies for 1 h at room temperature. ECL chemiluminescence kit (Millipore) and LAS‐3000 detection system were used to visualize the target protein band. For details of antibodies, see Table S3.

### 4.14 Chromatin Immunoprecipitation‐qPCR

Chromatin immunoprecipitation (ChIP) was performed using the BeyoChIP Assay Kit (Beyotime, P2080S) following the manufacturer's instructions. Briefly, approximately 1 × 10^6^ cells were cross‐linked with formaldehyde for 10 min at 37°C, and the chromatin was subsequently sheared by ultrasonic sonication. The resulting chromatin solution was diluted and incubated overnight at 4°C with the indicated primary antibody under gentle rotation, while normal IgG served as a negative control. Immune complexes were captured with Protein A/G magnetic beads for 1 h at 4°C, washed, and eluted to recover the bound DNA. The purified DNA fragments were analyzed by ChIP‐PCR or ChIP‐qPCR using specific primers targeting the promoter regions of interest (Table S4).

### 4.15 Dual‐Luciferase Reporter Assay

HT22 cells were co‐transfected with the mouse *Pdcd1* promoter‐firefly luciferase reporter plasmid, pRL‐TK Renilla luciferase plasmid, and empty vector, Flag‐H2B‐WT, or Flag‐H2B‐K120R plasmid using Lipofectamine 2000. After transfection, cells were treated with LPS for 24h. Firefly and Renilla luciferase activities were measured using a dual‐luciferase reporter assay system according to the manufacturer's instructions (Vazyme, DL101‐01). Firefly luciferase activity was normalized to Renilla luciferase activity, and the normalized values were further expressed relative to the EV+Ctrl group, which was set to 1.

### 4.16 In Vitro Deacylation Assay

The in vitro deacylation assay was performed by incubating synthetic H2BK120su peptide (1 µg, Shanghai Apeptide Co., Ltd) with recombinant human SIRT7 protein (ab104032, Abcam, Cambridge, UK) in deacylation reaction buffer containing 50 mM Tris‐HCl, 150 mM NaCl, and 1 mM DTT, pH 8.0. NAD^+^ (1.0 mM, IN0010, Solarbio) was added as the cofactor for the sirtuin enzymatic reaction. To determine NAD^+^ dependency, reactions were performed in the presence or absence of NAD^+^. To inhibit SIRT7‐mediated deacylation, nicotinamide (NAM; 10 mM, HY‐B0150, Merck) was added where indicated. Recombinant SIRT7 was added at the indicated doses, and the reactions were incubated for 3 h at 37°C. The samples were subsequently analyzed by dot blotting using an anti‐H2BK120su antibody. Recombinant SIRT7 input was verified by Western blotting.

### 4.17 Molecular Docking Analysis

For SIRT7‐H2B peptide docking, the catalytic core of human SIRT7 (residues 101–362; PDB ID: 9GMK) was used as the receptor. The structure was processed in ChimeraX by removing non‐protein molecules and disordered regions, and then prepared in AutoDockTools 1.5.7 by adding polar hydrogens and Gasteiger charges. A heptapeptide corresponding to histone H2B residues 117–123 with a K121E substitution was used as the ligand. This lysine corresponds to the H2BK120 site analyzed in our study, and the K‐to‐E substitution was used as a charge‐mimetic surrogate for lysine succinylation. Docking was performed using AutoDock Vina 1.2.7 with a grid box covering the catalytic cleft around Ser111 and His187. The exhaustiveness was set to 16, with 20 output modes and an energy range of 4 kcal/mol. A low‐energy pose spanning the inter‐lobe groove was selected for interaction analysis.

For PD‐L1‐PINK1 docking, the human PINK1 kinase domain was docked to the PD‐L1 cytoplasmic tail (residues 260–290) using the HADDOCK 2.4 web server. Acidic residues on the cytosolic surface of the PINK1 C‐lobe and the PD‐L1 cytoplasmic tail were defined as active residues, and docking was performed using default sampling and scoring parameters. The best‐scoring cluster was selected for further analysis. Representative models were visualized in ChimeraX, and interface residues were defined as those with any heavy atom within 4 Å of the partner chain. Hydrogen bonds, salt bridges, and hydrophobic contacts were analyzed using ChimeraX and LigPlot+ v2.3.

### 4.18 Mitochondrial Isolation

Mitochondria were isolated from HT22 cells using a mitochondrial isolation kit (Beyotime Biotechnology, C3601) according to the manufacturer's instructions. The cytosolic and mitochondrial fractions were subsequently collected and subjected to Western blot analysis.

### 4.19 Co‐Immunoprecipitation (Co‐IP)

Co‐immunoprecipitation assays were performed to examine protein‐protein interactions in HT22 cells. Cells were lysed in ice‐cold lysis buffer supplemented with protease inhibitors. Equal amounts of protein lysates were incubated overnight at 4°C with the indicated primary antibodies, including anti‐PINK1, anti‐PD‐L1, anti‐H2BK120su, or anti‐SIRT7 antibodies. Immune complexes were captured using protein A/G agarose beads, extensively washed, and eluted by boiling in SDS loading buffer. The precipitated proteins were subsequently analyzed by Western blot using the corresponding antibodies to assess the interactions between PINK1 and PD‐L1, as well as H2BK120su and SIRT7, in a reciprocal manner.

For the PD‐L1 cytoplasmic‐tail deletion assay, HT22 cells were transfected with si‐*Cd274* to silence endogenous PD‐L1 and then reconstituted with PD‐L1 WT or PD‐L1 ΔCT plasmids, followed by LPS stimulation. Cell lysates were immunoprecipitated with anti‐PD‐L1 antibody or control IgG and immunoblotted with anti‐PINK1 and anti‐PD‐L1 antibodies.

For the H2B‐K120 mutation assay, HT22 cells were transfected with empty vector, Flag‐H2B‐WT, or Flag‐H2B‐K120R plasmids. Cell lysates were immunoprecipitated with anti‐Flag antibody or control IgG, followed by immunoblotting with anti‐Flag, anti‐H2BK120su, anti‐H2B, and anti‐SIRT7 antibodies.

### 4.20 In Situ Proximity Ligation Assay (PLA)

The interaction between PD‐L1 and PINK1 in HT22 cells was assessed using the NaveniFlex Cell Red PLA Kit (Navinci Diagnostics, Cat. No. 60025) according to the manufacturer's instructions with minor optimization. Briefly, cells were seeded on glass coverslips and fixed with 4% paraformaldehyde for 15 min at room temperature, followed by permeabilization with 0.1% Triton X‐100 in PBS for 10 min. After blocking, cells were incubated simultaneously with mouse anti‐PD‐L1 and rabbit anti‐PINK1 primary antibodies at 4°C overnight.

The next day, cells were washed and incubated with the NaveniFlex Cell Red Navenibody probes (anti‐mouse and anti‐rabbit oligo‐conjugated secondary probes) according to the kit protocol. Ligation and rolling‐circle amplification steps were performed to generate fluorescent signals at sites of proximity between PD‐L1 and PINK1. Following amplification, nuclei were counterstained with DAPI, and fluorescence images were acquired with a confocal microscope. PLA signals (red fluorescent puncta) were quantified per cell to determine the relative interaction levels of PD‐L1 and PINK1.

### 4.21 Reverse Transcription and Quantitative Polymerase Chain Reaction (RT‐qPCR)

RNA isolation and RT‐qPCR were performed. Briefly, RNA was extracted by Trizol reagent, and 1 µg total RNA was used for cDNA synthesis and reversed transcribed by ReverTra Ace kit (TOYOBO). Then, the cDNA was used to quantify by the lightcycle480 system (Roche) with 2 × Power SYBRgreen mix (Applied Biosystems, Carlsbad, CA, United States). Finally, quantification of gene expression was calculated by normalization to GAPDH or Β‐ACTIN using the 2‐ΔCt method. Primer sequences for qPCR are shown in Table S5.

### 4.22 Transmission Electron Microscopy (TEM)

Hippocampus tissue and HT22 cell specimens were fixed in 2.5% glutaraldehyde, and 1% osmium acid, and then fixed and cut into ultrathin sections. The same fixation method was applied to the cell samples prepared for transmission electron microscopy. The samples were then stained with uranyl acetate and lead citrate and photographed by a Hitachi H7650 microscope (Tokyo, Japan). The ultrastructural analysis focused on the number and morphology of mitochondria undergoing mitophagy in hippocampal neurons. TEM observations in vivo and in vitro experiments were validated in three independent experiments. In each independent sample, mitochondria undergoing mitophagy were quantified from three randomly selected fields of view. Representative images showcasing typical features were chosen for presentation in the results.

### 4.23 Mitochondrial Membrane Potential (MMP) Measurement

The mitochondrial membrane potential (MMP) was evaluated using a JC‐1 assay kit (Solarbio, M8650) following the manufacturer's protocol. Briefly, HT22 cells were incubated with the JC‐1 working solution at 37°C for 20 min, then gently rinsed twice with PBS to remove excess dye. Fluorescence images were captured under an inverted fluorescence microscope. Mitochondria exhibiting intact membrane potential emitted red fluorescence due to JC‐1 aggregation, whereas depolarized mitochondria with reduced MMP displayed green fluorescence from JC‐1 monomers.

### 4.24 Intracellular ATP Level Measurement

Intracellular ATP content was measured using an Enhanced ATP Assay Kit (Beyotime Biotechnology, S0027) according to the manufacturer's instructions. Briefly, cells were washed once with PBS and lysed using the provided lysis buffer. The supernatant was then collected and mixed with the luciferase working solution, and the resulting luminescence intensity was immediately recorded using a microplate reader. ATP concentrations were calculated based on an ATP standard curve and normalized to the protein content of each sample, which was determined using the BCA Protein Assay Kit.

### 4.25 Novel Object Recognition Test (NORT)

The test was started on the 7th day after molding. The novel object recognition test was performed to assess recognition memory in mice. The test was conducted in a white plastic open‐field box (50 cm × 50 cm × 40 cm). Each mouse was placed in the arena containing two identical objects and allowed to freely explore for 5 min during the training session. After a 4‐h interval, one familiar object was replaced with a novel object differing in color, shape, and size. Mice were again allowed to explore for 5 min, and their behavior was recorded using a video‐tracking system. The recognition index was calculated as the number of novel‐object explorations divided by the total explorations of both objects, and the exploration ratio was defined as the time spent exploring the novel object divided by the total exploration time. The apparatus and objects were cleaned with 75% ethanol between trials to remove odor cues.

### 4.26 Y‐Maze Spontaneous Alternation Test

On the second day after the NORT, the Y‐maze spontaneous alternation test was performed to evaluate spatial working memory in mice. The maze (Shanghai Xinsoft Information Technology Co., China) consisted of three identical arms arranged at 120° angles. Each mouse was placed at the end of one arm and allowed to freely explore all three arms for 5 min. The sequence and total number of arm entries were automatically recorded using a video tracking system. An arm entry was defined as the entry of all four paws into an arm. A spontaneous alternation was defined as consecutive entries into three different arms, regardless of order (e.g., ABC, BCA, or CAB). The percentage of spontaneous alternation was calculated as:

$$Alternation\;\left(\%\right)=\frac{Number\;of\;alternations}{Total\;arm\;entries\;-\;2}\times 100\%$$

Total arm entries were also recorded to evaluate locomotor and exploratory activity. Between animals, the maze was cleaned with 75% ethanol to eliminate residual odor cues.

### 4.27 Statistical Analysis

Statistical analysis of all experiments was performed using GraphPad Prism 9 software (La Jolla, CA, USA). Data were presented as the mean ± standard error from three independent experiments. Student's t‐test and One‐way ANOVA followed by Tukey's test were used for comparison. Statistical parameters can be found in the numbers and graphical legends. Significant were set as p < 0.05.

## Author Contributions

**Weidong Gu**, **Lina Huang**, and **Liangfang Yao** were responsible for conceptualization and study design. **Na Meng**, **Jiateng Zhou**, and **Xiaoyu Guo** contributed to the experiments, data analysis and manuscript composition. **Ting Hong**, **Xuelian Li**, **Xingyu Wei**, **Chanhua Zhang**, **Xixue Zhang**, **Songbin Liu**, and **Yulin Zhang** performed the in vivo experiments. All authors reviewed and approved the final version of the manuscript.

## Conflicts of Interest

The authors declare no conflicts of interest.

## Supporting information




**Supporting File 1**: advs77642‐sup‐0001‐SuppMat.docx.

**Supporting File 2**: advs77642‐sup‐0002‐SuppMat.zip.

## Data Availability

The data that support the findings of this study are available from the corresponding author upon reasonable request.
