# Supplementary material for: SIRT7‐Mediated H2BK120 Succinylation Drives Aberrant Mitophagy in Sepsis‐Associated Cognitive Dysfunction
Source: Adv Sci (Weinh). 2026 Sep 8:e77642. Online ahead of print. doi: 10.1002/advs.77642 (PMC13554371; doi:10.1002/advs.77642)
Supplement: Supplementary file 1 [file ADVS-9999-e77642-s001.docx]

Fig S1. Untargeted metabolomic analysis of hippocampal tissues from CLP mice.

a. Comparison of TICs of QC samples in positive-ion mode (left) and negative-ion mode (right).
b. PCA of identified metabolites showing clustered distribution of QC samples, n = 8 mice per group.
c. Number of metabolites detected in positive- and negative-ion modes.
d. Proportion of identified metabolites classified into different chemical categories, including benzenes and substituted derivatives, carboxylic acids and derivatives, fatty acyls, and glycerophospholipids.
e. PCA of identified metabolites.
f. Volcano plot of differentially expressed metabolites (DEMs) between septic and control groups. Upregulated metabolites in sepsis are shown in red, and downregulated metabolites are shown in blue.
g. Pathway analysis of upregulated (left) and downregulated (right) metabolites.

FigS2. LPS-induced protein succinylation in hippocampal tissues and HT22 cells.

a. Bar chart showing the proportion of differentially abundant intracellular metabolites by category.

b. Representative images of Ksucc staining in Iba-1-positive microglia in hippocampal tissues from control and CLP mice 24 hours after surgery. Scale bar, 50 μm, n = 3 mice per group.

c. Representative images of Ksucc staining in GFAP-positive astrocytes in hippocampal tissues from control and CLP mice 24 hours after surgery. Scale bar, 50 μm, n = 3 mice per group.

d. Representative immunofluorescence images and quantitative analysis (e) of Ksucc staining in HT22 cells after 24 hours of LPS stimulation (50 μg/mL). Scale bar, 100 μm, n = 3 independent cell culture experiments.

f. Representative western blot analysis showing Ksucc levels in HT22 cells pretreated with DES (10 or 20 mM) for 3 hours before LPS stimulation, n = 3 independent cell culture experiments. DES, diethyl succinate. Data are shown as mean ± SD. Data in panel e were analyzed using unpaired two-tailed Student’s t-test. **p < 0.01.

FigS3. SP confers neuroprotection while suppressing protein succinylation.

a. Representative immunofluorescence staining images and quantitative analysis (b) showing Ksucc expression in HT22 cells pretreated with SP (50 μM) for 3 hours before LPS stimulation for 24 h. Scale bar, 100 μm, n = 3 independent cell culture experiments.

c. Representative immunofluorescence staining images and quantitative analysis (d) showing H2BK120su expression in HT22 cells pretreated with SP (50 μM) for 3 hours before LPS stimulation for 24 h. Scale bar, 20 μm, n = 3 independent cell culture experiments.

e. Succinyl-CoA levels in HT22 cell lysates after LPS stimulation for 24 h with or without SP treatment or *Ogdh* knockdown. Succinyl-CoA concentrations were measured by ELISA and normalized to total protein content, n = 3 independent cell culture experiments.

f. Representative western blot analysis of H2BK120su levels in HT22 cells treated with LPS with or without *Ogdh* knockdown, n = 3 independent cell culture experiments.

g. Representative PI staining images of HT22 cells treated with LPS with or without *Ogdh* knockdown. Red fluorescence indicates PI-positive dead cells. Scale bar, 400 μm, n = 3 independent cell culture experiments.

h. Representative images of TUNEL staining and quantitative analysis (i) showing apoptotic hippocampal neurons in CLP mice pretreated with SP (0.5 mg/kg). Scale bar, 50 μm, n = 3 mice per group. Data are shown as mean ± SD. Data in panels b, d-e, and i were analyzed by one-way ANOVA followed by Dunnett’s multiple comparisons test. *p < 0.05, **p < 0.01, ***p < 0.001 and ****p < 0.0001; ns, not significant.

FigS4. Mitophagy is increased in hippocampal neurons of CLP mice.

a.Heatmap showing differentially expressed genes (DEGs) in hippocampal tissues of sham and CLP mice.

b.GO analysis reveals significant differences in biological processes.

c.KEGG pathway enrichment analysis of DEGs identified in hippocampal tissues from sham and CLP mice.

d.Top 20 KEGG pathways enriched for alternative splicing (AS) events identified in hippocampal tissues from sham and CLP mice.

FigS5. Excessive PINK1-Parkin-dependent mitophagy contributes to neuronal death in the hippocampus of CLP mice and LPS-treated HT22 cells.

a. Western blot analysis showing the expression of LC3-II/I, PINK1, Parkin, SQSTM1 (p62), and TOM20 in hippocampal tissues of Ctrl and CLP mice. Each lane represents one mouse; n = 3 mice per group.

b. Quantitative analysis of LC3-II/I, SQSTM1 (p62), TOM20, PINK1 and Parkin in hippocampal tissues of Ctrl and CLP mice.

c. Quantitative analysis of LC3-II/I, PINK1, Parkin, and SQSTM1 (p62) expression in hippocampal tissues of Ctrl, CLP, and CLP+SP mice.

d. Western blot analysis and quantitative analysis (e) showing the expression of LC3-II/I, PINK1, Parkin, SQSTM1 (p62), and TOM20 in control and LPS-stimulated HT22 cells, n = 3 independent cell culture experiments.

f. Representative PI staining images and quantitative analysis (g) showing cell death in HT22 cells treated with Mdivi-1 (25 μM) or CQ (20 μM) for 2 hours following LPS stimulation. Scale bar, 400 μm, n = 3 independent cell culture experiments.

h. Representative western blot analysis showing LC3-II/I and SQSTM1 (p62) expression in hippocampal tissues from mice treated with CQ (75 or 150 mg/kg) 18 hours after CLP surgery and collected 6 hours later, n = 3 mice per group.

i. Representative fluorescence images and quantitative analysis (j) of DCFH-DA staining showing intracellular ROS levels in HT22 cells treated with SP (50 μM) or Mdivi-1 (25 μM) for 2 hours following LPS stimulation. Scale bar, 100 μm, n = 3 independent cell culture experiments.

k. Representative TUNEL fluorescence images and quantitative analysis (l) of the hippocampal CA3 region in CLP mice pretreated with Mdivi-1 (1.5 mg/kg) 12 hours before surgery or treated with CQ (150 mg/kg) 6 hours before tissue collection. Hippocampal tissues were collected and analyzed 24 hours after CLP surgery. Scale bar, 50 μm, n = 3 mice per group. Data are shown as mean ± SD. For animal experiments, n indicates the number of mice per group; for HT22 cell experiments, n indicates independent cell culture experiments. Data in panels b and e were analyzed using unpaired two-tailed Student’s t-test. Data in panels c, g, j, and l were analyzed by one-way ANOVA followed by Dunnett’s multiple comparisons test. *p < 0.05, **p < 0.01, ***p < 0.001 and ****p < 0.0001; ns, not significant.

FigS6. Inhibition of the PD-1/PD-L1 signaling axis alleviates excessive mitophagy activation.

a. *Pdcd1* promoter activity was assessed by dual-luciferase reporter assay in HT22 cells co-transfected with *Pdcd1* promoter-firefly reporter, pRL-TK Renilla luciferase, and empty vector, Flag-H2B-WT, or Flag-H2B-K120R. Firefly luciferase activity was normalized to Renilla luciferase activity and expressed relative to the EV+Ctrl group, n = 3 independent cell culture experiments.

b. qRT-PCR analysis of *Pdcd1*, *Tmem52*, and *Cd274* mRNA levels in hippocampal tissues, n = 3 mice per group.

c. Western blot and quantitative analysis (d) showing PD-1 and PD-L1 protein expression levels in hippocampal tissues of Ctrl and CLP mice. Each lane represents one mouse; n = 3 mice per group.

e. Representative immunofluorescence images and quantitative analysis (f) showing PD-L1 expression in NeuN-positive neurons in the hippocampal region of Ctrl and CLP mice. Scale bar, 50 μm, n = 3 mice per group.

g. Western blot analysis and quantitative analysis (h) showing PD-1 and PD-L1 protein expression levels in HT22 cells, n = 3 independent cell culture experiments.

i. Representative western blot analysis confirming *Pdcd1* knockdown in HT22 cells transfected with *Pdcd1* shRNA, n = 3 independent cell culture experiments.

j. Representative confocal immunofluorescence images illustrating the co-localization of PD-L1 with MitoTracker signals in HT22 cells. Scale bars, 20 μm (left) and 10 μm (right), n = 3 independent cell culture experiments.

k. Representative Co-IP assay showing the physical interaction between PINK1 and PD-L1 proteins in HT22 cells, n = 3 independent cell culture experiments.

l. Representative Co-IP assay showing the interaction between PINK1 and PD-L1 proteins in HT22 cells following LPS stimulation, n = 3 independent cell culture experiments.

m. Western blot analysis and quantitative analysis (n) showing LC3-II/I and SQSTM1 (p62) protein levels in HT22 cells following treatment with LPS, PD-1 neutralizing antibody (10 μg/mL), or PD-L1 neutralizing antibody (10 μg/mL), n = 3 independent cell culture experiments.

o. Representative confocal immunofluorescence images and quantitative analysis (p) illustrating the co-localization of LC3-II with MitoTracker signals in HT22 cells. Scale bars, 15 μm (left) and 5 μm (right), n = 3 independent cell culture experiments.

q. Representative fluorescence images and quantitative analysis (r) of PI staining in HT22 cells following the indicated treatments. Scale bar, 400 μm, n = 3 independent cell culture experiments. Data are shown as mean ± SD. For animal experiments, n indicates the number of mice per group; for HT22 cell experiments, n indicates independent cell culture experiments. Data in panel a were analyzed using one-way ANOVA followed by Šídák’s multiple comparisons test for selected pairwise comparisons. Data in panels b, d, f, and h were analyzed using unpaired two-tailed Student’s t-test. Data in panels m, p, and r were analyzed by one-way ANOVA followed by Dunnett’s multiple comparisons test. *p < 0.05, **p < 0.01, ***p < 0.001 and ****p < 0.0001; ns, not significant.

FigS7. The desuccinylase SIRT7 is markedly downregulated in the hippocampus of CLP mice and in LPS-stimulated HT22 cells.

a. qRT-PCR analysis showing the mRNA expression levels of key enzymes associated with the regulation of protein succinylation in hippocampal tissues, n = 3 mice per group.

b. Representative images and quantitative analysis of SIRT7 immunostaining in HT22 cells. Scale bar, 20 μm, n = 3 independent cell culture experiments.

c. Representative Co-IP analysis of the interaction between H2B and SIRT7 in HT22 cells. Cells were transfected with EV, Flag-H2B-WT, or Flag-H2B-K120R, and cell lysates were immunoprecipitated with anti-Flag or control IgG followed by immunoblotting with the indicated antibodies. Both H2B-WT and H2B-K120R carried the same Flag tag. Intervening marker lanes were removed and indicated by vertical lines, n = 3 independent cell culture experiments.

d. Representative western blot analysis showing Ksucc levels in LPS-stimulated HT22 cells after overexpression of *Suclg1*, *Suclg2*, or *Sucla2*, n = 3 independent cell culture experiments.

e. Representative western blot analysis showing SIRT7 protein expression in HT22 cells following SIRT7 overexpression, accompanied by a schematic illustration of the transfected plasmid, n = 3 independent cell culture experiments.

f. Kaplan-Meier survival analysis of mice after AAV-*Sirt7* overexpression in the hippocampal CA3 region, n = 12 mice per group.

g. Western blot analysis showing SIRT7 protein expression in the hippocampus after CA3-targeted AAV-*Sirt7* overexpression. Each lane represents one mouse; n = 3 mice per group.

h. Representative fluorescence images illustrating viral expression in the hippocampal CA3 region after AAV-mediated *Sirt7* overexpression. Scale bar, 100 μm, n = 3 mice per group. AAV, adeno-associated virus; EV, empty vector. Data are shown as mean ± SD. For animal experiments, n indicates the number of mice per group; for HT22 cell experiments, n indicates independent cell culture experiments. Data in panels a and b were analyzed using unpaired two-tailed Student’s t-test. Data in f were analyzed by the log-rank test. *p < 0.05 and **p < 0.01; ns, not significant.

FigS8. Neuron-specific SIRT7 deletion exacerbates CLP-induced succinylation in the mouse hippocampus.

a. Western blot analysis showing the expression levels of Ksucc and H2BK120su in hippocampal tissues. Each lane represents one mouse; n = 3 mice per group.

b. Western blot analysis showing the expression levels of global lysine acetylation (Kac) and site-specific H2BK120 acetylation (H2BK120ac) in hippocampal tissues. Each lane represents one mouse; n = 3 mice per group.

c. Representative TUNEL fluorescence images and quantitative analysis (d) of the hippocampal CA3 region in CLP mice pretreated with SP. Scale bar, 50 μm, n = 3 mice per group.

e. Representative immunofluorescence images and quantitative analysis (f) showing PD-L1 expression in NeuN-positive neurons in the hippocampal CA3 region. Scale bar, 50 μm, n = 3 mice per group.

g. Representative immunofluorescence images showing co-localization of PINK1 and PD-L1 in the hippocampal CA3 region. Scale bar, 10 μm, n = 3 mice per group.

h. Quantitative analysis of the cytoplasmic volume fraction of mitophagic vacuoles in hippocampal neurons from WT+CLP, cKO+CLP, and cKO+CLP+SP mice, n = 3 mice per group.

i. Quantitative analysis of the cytoplasmic volume fraction of mitophagic vacuoles in hippocampal neurons from cKO+CLP mice treated with intra-HPC IgG or anti-PD-L1 antibody, n = 3 mice per group.

j. Representative TEM images illustrating synaptic ultrastructure in WT+CLP, cKO+CLP, and cKO+CLP+SP mice. Scale bar, 100 nm, n = 3 mice per group.

k. Representative TEM images illustrating synaptic ultrastructure in cKO+CLP mice treated with intra-HPC IgG or anti-PD-L1 antibody. Scale bar, 100 nm, n = 3 mice per group.

l. Quantitative analysis of synaptic cleft width and PSD thickness in WT+CLP, cKO+CLP, and cKO+CLP+SP mice.

m. Quantitative analysis of synaptic cleft width and PSD thickness in cKO+CLP mice treated with intra-HPC IgG or anti-PD-L1 antibody. Data are shown as mean ± SD. For animal experiments, n indicates the number of mice per group. Data in i and m were analyzed using unpaired two-tailed Student’s t-test. Data in d, f, h, and l were analyzed by one-way ANOVA followed by Dunnett’s multiple comparisons test. *p < 0.05, **p < 0.01, ***p < 0.001 and ****p < 0.0001; ns, not significant.

FigS9. Overexpression of SIRT7 significantly alleviates mitochondrial oxidative stress.

a. Quantitative analysis of SIRT7, H2BK120su, PD-1, and PD-L1 protein levels in HT22 cells after SIRT7 overexpression, n = 3 independent cell culture experiments.
b. Quantitative analysis of mitophagy-related proteins (LC3-II/I, PINK1, Parkin, SQSTM1/p62, and TOM20) in HT22 cells after SIRT7 overexpression, n = 3 independent cell culture experiments.
c. Quantitative analysis of ATP production in HT22 cells, n = 3 independent cell culture experiments.
d. Representative fluorescence images and quantitative analysis (e) of JC-1 staining in HT22 cells after SIRT7 overexpression. Scale bar, 100 μm, n = 3 independent cell culture experiments.

f. Representative fluorescence images and quantitative analysis (g) illustrating DCFH-DA-detected reactive oxygen species (ROS) levels in HT22 cells after SIRT7 overexpression. Scale bar, 100 μm, n = 3 independent cell culture experiments.

h. Quantitative analysis of H2BK120su, PD-1/PD-L1 signaling components, and mitophagy-related proteins (LC3-II/I, PINK1, Parkin, SQSTM1/p62, and TOM20) in the hippocampus after CA3 injection of AAV-Sirt7-OE, n = 3 mice per group. Data are shown as mean ± SD. For animal experiments, n indicates the number of mice per group; for HT22 cell experiments, n indicates independent cell culture experiments. Data in panels a-c, e, and g-h were analyzed by one-way ANOVA followed by Dunnett’s multiple comparisons test. *p < 0.05, **p < 0.01, ***p < 0.001 and ****p < 0.0001; ns, not significant.

FigS10. SIRT7 modulates mitochondrial ROS generation and apoptotic cell death in HT22 cells.

a. Representative fluorescence images and quantitative analysis (b) illustrating ROS levels detected by DCFH-DA in HT22 cells after pretreatment with RSV or a SIRT7 inhibitor. Scale bar, 100 μm, n = 3 independent cell culture experiments.

c. Representative TUNEL fluorescence images and quantitative analysis (d) showing apoptotic HT22 cells after pretreatment with RSV or a SIRT7 inhibitor. Scale bar, 200 μm, n = 3 independent cell culture experiments. Data are shown as mean ± SD. For HT22 cell experiments, n indicates independent cell culture experiments. Data in panels b and d were analyzed by one-way ANOVA followed by Dunnett’s multiple comparisons test. *p < 0.05, **p < 0.01, and ***p < 0.001; ns, not significant.

**Supplementary Table 1: Target sequence for shRNA and siRNA**

| Name | Target gene | Type | Target region | Sequence (5’-3’) |
| --- | --- | --- | --- | --- |
| sh-*Sirt7*#1 | *Sirt7* | shRNA | CDS | GACATGAGGATGGACATTGTT |
| sh-*Sirt7*#2 | *Sirt7* | shRNA | CDS | CAGGCATGACTTCCACATGAA |
| sh-*Sirt7*#3 | *Sirt7* | shRNA | CDS | GCTCGTGGTAACAGAGAGAAT |
| si-*Cd274* | *Cd274* | siRNA | 3’UTR | sense: GGUAAGAAUUUGUCGUUAAUU; antisense: UUAACGACAAAUUCUUACCTT |
| si-*Ogdh* | *Ogdh* | siRNA | CDS | sense: GAAGCCAACUUCGACAUAATT  antisense: UUAUGUCGAAGUUGGCUUCTT |

**Supplementary Table 2. Plasmids used for in vitro studies**

| **Plasmid** | **Target gene** | **Species** | **Purpose** | **Backbone** | **Promoter** | **Tag/**  **Reporter** | **Selection marker** |
| --- | --- | --- | --- | --- | --- | --- | --- |
| pRP[Exp]-EF1A-*mSirt7*-FLAG | *Sirt7* | Mouse | Overexpression | pRP[Exp] | EF1A | FLAG | EGFP / Puromycin |
| pRP[shRNA]-U6-*mPdcd1*-shRNA | *Pdcd1* | Mouse | Knockdown | pRP[shRNA] | U6 (shRNA) / hPGK (reporter) | EGFP | Puromycin |
| pLV3-CMV-*Sucla2*(mouse)-3×FLAG-Puro | *Sucla2* | Mouse | Overexpression | pLV3 | CMV | 3×FLAG | Puromycin |
| pLV3-CMV-*Suclg2*(mouse)-3×FLAG-Puro | *Suclg2* | Mouse | Overexpression | pLV3 | CMV | 3×FLAG | Puromycin |
| pLV3-CMV-*Suclg1*(mouse)-3×FLAG-Puro | *Suclg1* | Mouse | Overexpression | pLV3 | CMV | 3×FLAG | Puromycin |
| pcDNA3.1(+)-mPD-L1-WT | *Cd274* | Mouse | WT rescue after *Cd274* knockdown | pcDNA3.1(+) | CMV | None | Not used |
| pcDNA3.1(+)-mPD-L1-ΔCT | *Cd274* | Mouse | ΔCT rescue after *Cd274* knockdown; aa 1–260 | pcDNA3.1(+) | CMV | None | Not used |
| Flag-H2B-WT | Histone H2B | Mouse | H2B WT overexpression | pcDNA3.1(+) | CMV | N-terminal 3×FLAG | Not used |
| Flag-H2B-K120R | Histone H2B | Mouse | H2B-K120R mutant overexpression | pcDNA3.1(+) | CMV | N-terminal 3×FLAG | Not used |
| pGL3-*Pdcd1* promoter | *Pdcd1* | Mouse | Firefly luciferase reporter assay | pGL3-Basic | *Pdcd1* promoter | Firefly luciferase | Not used |
| pRL-TK | — | — | Internal control for dual-luciferase assay | pRL-TK | TK | Renilla luciferase | Not used |

**Supplementary Table 3: Details of Antibodies**

| **Antibodies** | **SOURCE** | **IDENTIFIER** |
| --- | --- | --- |
| Anti-Succinyllysine | PTM BIO | Cat# PTM-401 |
| Anti-Succinyl-Histone H2B (Lys120) | PTM BIO | Cat# PTM-409 |
| Anti-Succinyl-Histone H3 (Lys122) | PTM BIO | Cat# PTM-413 |
| Anti-Succinyl-Histone H3 (Lys79) | PTM BIO | Cat# PTM-412 |
| Anti-Succinyl-Histone H3 (Lys14) | PTM BIO | Cat# PTM-421 |
| Anti-Acetyl-Histone H2B (Lys120) | PTM BIO | Cat# PTM-111 |
| Anti-Acetyllysine | PTM BIO | Cat# PTM-132 |
| Anti-Histone H2B | Cell Signaling Technology | Cat# 12364T |
| Anti-Histone H3 | PTM BIO | Cat# PTM-6613 |
| Anti-SUCLA2 | PTM BIO | Cat# PTM-3126 |
| Anti-SUCLG2 | PTM BIO | Cat# PTM-3560 |
| Anti-SUCLG1 | Proteintech | Cat# 14923-1-AP |
| Anti-SIRT7 | Proteintech | Cat# 12994-1-AP |
| Anti-SIRT5 | Proteintech | Cat# 15122-1-AP |
| Anti-PINK1 | Proteintech | Cat# 23274-1-AP |
| Anti-PINK1 | SANTACRUZ | Cat# sc-517353 |
| Anti-LC3B | Abcam | Cat# ab192890 |
| Anti-PARK2 | Proteintech | Cat# 66674-1-Ig |
| Anti-P62/SQSTM1 | Proteintech | Cat# 18420-1-AP |
| Anti-TOM20 | Proteintech | Cat# 11802-1-AP |
| Anti-PD-1 | Abcam | Cat# ab300425 |
| Anti-PD-L1 | Abcam | Cat# ab213480 |
| Anti-PD-1 | Selleck | Cat# A2122 |
| Anti-PD-L1 | Selleck | Cat# A2115 |

**Supplementary Table 4: Primers for ChIP-qPCR, related to Figure 4.**

| **RNA name** | **Sequences (5’-3’)** |
| --- | --- |
| Mouse-*Pdcd1*-Forward① | CCCACACATCTCATTGCTAATA |
| Mouse-*Pdcd1*-Reverse① | GTACCTGGCTGGCTAGTCAT |
| Mouse-*Pdcd1*-Forward② | CCTGGTGTTAGGTTTTTCTCA |
| Mouse-*Pdcd1*-Reverse② | TCTCACTGTTTTCTCTGCTTCTA |
| Mouse-*Pdcd1*-Forward③ | CACAAGAGGGGCTAGAAATG |
| Mouse-*Pdcd1*-Reverse③ | GGGGGGTTCTCTCTGACTA |

**Supplementary Table 5: List of qPCR primers**

| **Gene** | **Forward** | **Reverse** |
| --- | --- | --- |
| Mouse-*Sirt7* | CTGCTAGCAAAGCAGACACAATC | GGCTTGGAGGCTTCGTCAT |
| Mouse-*Sirt5* | ATCGCAAGGCTGGCACCAAGAA | CTAAAGCTGGGCAGATCGGACT |
| Mouse-*Pdcd1* | ATGACTTCCACATGAACATCCT | CTCCAGGATTCTCTCTGTTACC |
| Mouse-*Pdl1* | TGCGGACTACAAGCGAATCACG | CTCAGCTTCTGGATAACCCTCG |
| Mouse-*Tmem52* | ATCCCAGTGGACAGTGATAGCC | CTCAGACGCATAGAGGCTGTAG |
| Mouse-*Hdac1* | ACCATCAAAGGACACGCCAAG | CCACCGCTGTTTCGTAAGTCC |
| Mouse-*Hdac2* | GTTTTGTCAGCTCTCCACGGGT | CTTGGCATGATGTAGTCCTCCAG |
| Mouse-*Hdac3* | AACCTCATCGCCTGGCATTGAC | GTAGTCCTCAGAATGGAAGCGG |
| Mouse-*Hat1* | AAGATCCGTCCAGAAGCTATTTG | ACATTTGCTGTGCCTGTATTGC |
| Mouse-*Kat2α* | GGTCAAGGGCTATGGCACCC | GGTGAGAAAGTAAAGGATGCTGTG |
| Mouse-*Cpt1a* | GGCATAAACGCAGAGCATTCCTG | CAGTGTCCATCCTCTGAGTAGC |
| Mouse-*Cbp/p300* | CACCATCTGTGGCTACTCCTCA | GGTTTCAGCACTGGTCACAGAG |
| Mouse-*Dlst* | AGGTCTGCGTTCAGAACATCGG | AGGAAAGCATCCTTGTGCCGAG |
| Mouse-*Fh1* | GAATGGCAAGCCAAAATTCCTT | CGTTCTGTAGCACCTCCAATCTT |
| Mouse-*Suclg1* | GTCTTACACAGCCTCTCGGAAAC | ACTCCAAAGCCTGCTGACTGTG |
| Mouse-*Suclg2* | AGCTCAAGGTGCCACTGGTAGT | GCTTTCTTGGCTGCATCCTCCA |
| Mouse-*Sulca2* | TACTGATAGGAAGTGCACAAGGTG | CTCCTTTACAATGGCTTCAGGG |
| Mouse-*Gapdh* | CAAGAAGGTGGTGAAGCAGG | CCACCCTGTTGCTGTAGCC |
